# Fluorinated antimony(v) derivatives: strong Lewis acidic properties and application to the complexation of formaldehyde in aqueous solutions†
†Electronic supplementary information (ESI) available: Additional experimental and computational details and crystallographic data in cif format. CCDC 1483464–1483472. For ESI and crystallographic data in CIF or other electronic format see DOI: 10.1039/c6sc02558g



**DOI:** 10.1039/c6sc02558g

**Published:** 2016-07-11

**Authors:** Daniel Tofan, François P. Gabbaï

**Affiliations:** a Department of Chemistry , Texas A&M University , College Station , TX 77843 , USA . Email: francois@tamu.edu

## Abstract

As part of our ongoing studies of water tolerant Lewis acids, we have synthesized and investigated the properties of Sb(C_6_F_5_)_3_(O_2_C_6_Cl_4_), a fluorinated stiborane whose Lewis acidity approaches that of B(C_6_F_5_)_3_. While chloroform solutions of this Lewis acid can be kept open to air or exposed to water for extended periods of time, this new Lewis acid reacts with P^*t*^Bu_3_ and paraformaldehyde to form the corresponding formaldehyde adduct ^*t*^Bu_3_P–CH_2_–O–Sb(C_6_F_5_)_3_(O_2_C_6_Cl_4_). To test if this reactivity can also be observed with systems that combine the phosphine and the stiborane within the same molecule, we have also prepared *o*-C_6_H_4_(PPh_2_)(SbAr_2_(O_2_C_6_Cl_4_)) (Ar = Ph, C_6_F_5_). These yellow compounds, which possess an intramolecular P→Sb interaction, are remarkably inert to water but do, nonetheless, react with and accomodate formaldehyde into the P/Sb pocket. In the case of the fluorinated derivative *o*-C_6_H_4_(PPh_2_)(Sb(C_6_F_5_)_2_(O_2_C_6_Cl_4_)), formaldehyde complexation, which occurs in water/dichloromethane biphasic mixtures, is accompanied by a colourimetric turn-off response thus highlighting the potential that this chemistry holds in the domain of molecular sensing.

## Abstract 2

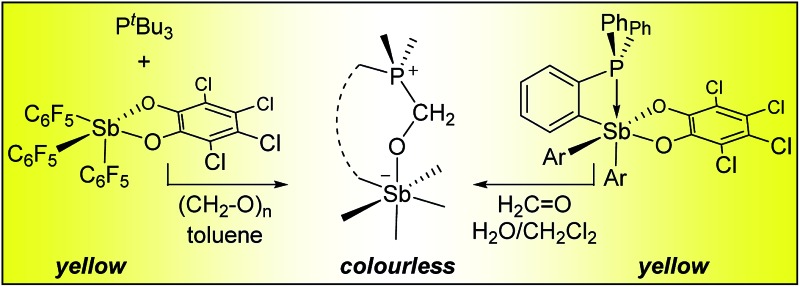
Lewis acidic fluorinated organoantimony(v) derivatives have been combined with phosphines for the complexation and colourimetric sensing of formaldehyde in biphasic water/CH_2_Cl_2_ mixtures.

## Introduction

Perfluorinated triarylboranes, such as B(C_6_F_5_)_3_ have become ubiquitous Lewis acids used in both organic and organometallic chemistry.^1–4^ These fluorinated organoboranes display uncompromised Lewis acidic properties that rival those of boron halides such as BCl_3_.^1,3^ However, because of the absence of reactive boron–halogen bonds, these boranes are not corrosive and tolerate air and moisture at least for short periods of time. Noteworthy applications for these fluorinated boranes include the activation of transition metal and main group species *via* anionic ligand abstraction.^1–5^ These fluorinated boranes have also been combined with bulky Lewis bases to generate frustrated Lewis pairs (FLPs) that have been shown to activate a wide variety of small molecules.^6–12^ While additional exciting applications may be discovered for the use of these boranes in organic media, their electron-deficiency, as well as the exposed nature of the boron centre, may preclude applications that require the use of water. To overcome this limitation and extend the use of main group Lewis acids to aqueous environments, we have recently begun a systematic investigation of other main group compounds that also display Lewis acidic properties.

**Fig. 1:**
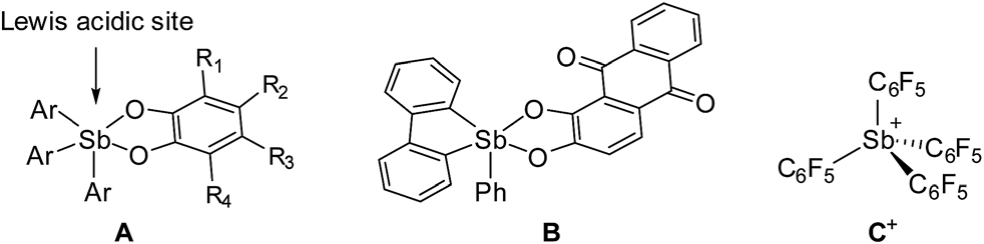
Selected examples of antimony(v) Lewis acids.

Recent efforts have shown that electrophilic phosphorus(v) compounds^13^ can be used as Lewis acid catalysts for organic reactions^14–18^ as well as in FLPs for hydrogenations^19^ and CO_2_ capture reactions.^20^ Inspired by these advances and using fluoride anion affinity data as a guide,^21,22^ several groups have paid a renewed attention to the properties of antimony(v) compounds.^23–26^ As part of our contribution to this area,^27–30^ we were drawn by the properties of simple neutral derivatives such as triaryl-catecholato-stiboranes (**A**, Fig. 1) which have been previously shown to form adducts with Lewis basic substrates.^31–34^ Building on these earlier studies, we synthesized and investigated additional examples of such compounds,^35^ including **B** (Fig. 1),^36^ and showed that they can be used for the complexation of fluoride anions under aqueous conditions. In parallel, we also considered the introduction of electron-withdrawing pentafluorophenyl substituents and reported the highly electrophilic stibonium cation **C^+^**(Fig. 1) which was too reactive for applications in aqueous media.^37^ With the view of identifying a compromise between high Lewis acidity and tolerance to water, we have now considered fluorinated versions of triaryl-catecholato-stiboranes of type **A**. In this article, we describe the properties of such Lewis acids and demonstrate that they can be combined with phosphines both intermolecularly and intramolecularly to display frustrated Lewis pair reactivity. The compatibility of these systems with aqueous media is illustrated by their use for the capture of formaldehyde in water.

## Results and discussion

### Synthesis and reactivity of a fluorinated stiborane

**Scheme 1:**
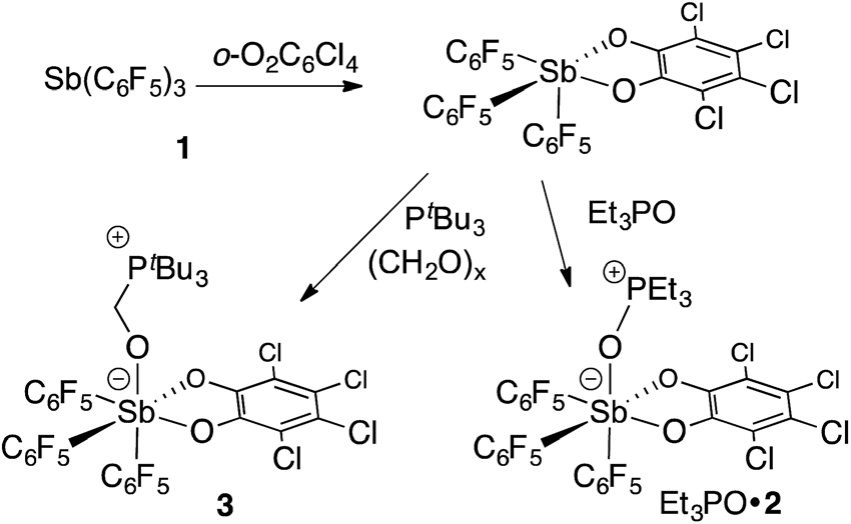
Preparation and reactivity of stiborane **2**.

In targeting easily accessible fluorinated stiboranes, we chose to attempt the oxidation of Sb(C_6_F_5_)_3_ (**1**).^38^ With *o*-chloranil, the oxidation is fast and selective, allowing isolation of the heteroleptic stiborane Sb(C_6_F_5_)_3_(O_2_C_6_Cl_4_) (**2**) in 69% yield as an analytically pure, orange, crystalline solid (Scheme 1). In solution, a single C_6_F_5_ environment is observed in the ^19^F NMR spectrum of chloroform solutions (Fig. 4b), even at –70 °C, thus indicating that the structure of this compound is fluxional. Stiborane **2** appears indefinitely stable in solutions kept open to atmospheric air, as no hydrolysis could be observed (^19^F NMR) upon layering of CDCl_3_ solutions of **2** with water.

**Fig. 2:**
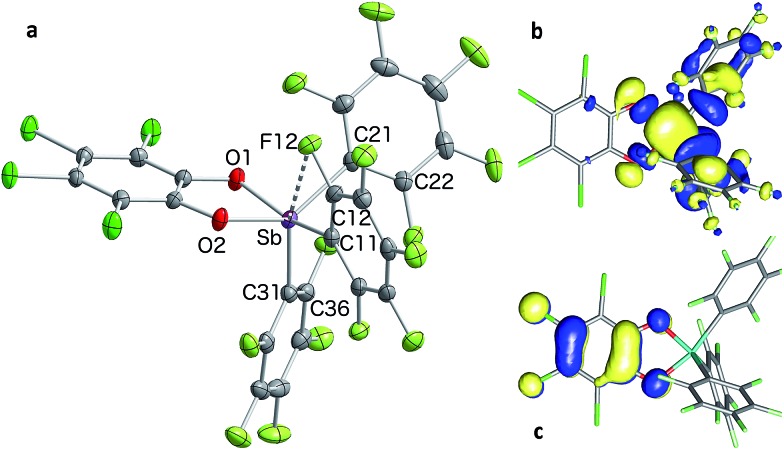
Solid-state structure of stiborane **2** with thermal ellipsoids drawn at the 50% probability level (a). Select solid-state distances [Å] and angles [°]: Sb–F(12) 3.0764(16), Sb–C(11) 2.145(2), Sb–C(21) 2.126(2), Sb–C(31) 2.106(2), Sb–O(1) 2.0321(15), Sb–O(2) 1.9951(15), O(2)–Sb–C(21) 138.53(7), O(1)–Sb–C(11) 157.75(7), C(31)–Sb–F(12) 157.39(6), Sb–C(11)–C(12) 116.25(15), Sb–C(21)–C(22) 119.94(16), Sb–C(31)–C(36) 120.66(16). The HOMO (c) and LUMO (b) are the only orbitals involved in the lowest computed excitation.

In the solid state, the molecule is frozen in a distorted square-pyramidal geometry at antimony (Fig. 2a), which is reminiscent of that observed for other triaryl-catecholato-stiboranes which have been used as Lewis acids.^35,36^ It is interesting to note that stiborane **2** exhibits a short Sb–F(12) contact of 3.0764(16) Å, which is well within the sum of the van der Waals radii of the two elements (∑_vdWR_(Sb,F) = 3.93 Å).^39^ The attractive nature of this Sb–F interaction is further supported by the value of the Sb–C(11)–C(12) angle of 116.25(15)°, which is compressed from the ideal value of 120°. The presence of the Sb–F(12) interaction is further supported by the fact that the Sb–C(21)–C(22) and Sb–C(31)–C(36) angles involving the other two C_6_F_5_ groups are much closer to 120° (119.94(16) and 120.66(16)°, respectively). While similar interactions have been observed in group 13 and 14 compounds,^40–48^ such short contacts are not observed in other known C_6_F_5_-decorated antimony(v) compounds, including Sb(C_6_F_5_)_5_.^37,49^

**Fig. 3:**
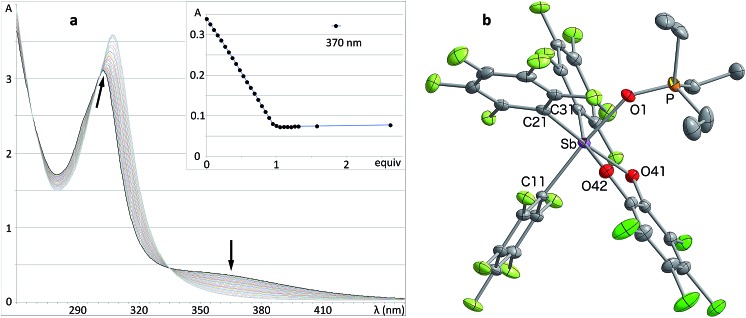
(a) UV titration of stiborane **2** with Et_3_PO showing the titration isotherm in inset. (b) Solid-state structure of Et_3_PO·**2**. Only one of the two independent molecules present in the asymmetric unit is shown. The thermal ellipsoids are drawn at the 50% probability level and the hydrogen atoms and solvent molecules are omitted for clarity. Select distances [Å] and angles [°] [the corresponding metrical parameters of the second independent molecule are given in brackets]: Sb–O(1) 2.107(2) [2.113(2)], P–O(1) 1.524(2) [1.510(2)], O(1)–Sb–C(11) 173.45(10) [174.35(11)], Sb–O(1)–P 143.33(15) [148.46(16)].

Solutions of stiborane **2** display an intense yellow colour in dichloromethane and chloroform as a result of an absorption band near 370 nm that tails into the visible range. However, in coordinating solvents such as acetone, methanol, THF, acetonitrile or DMF, the colour fades markedly suggesting the coordination of the solvent molecules to the antimony center. A similar effect is observed with water, which leads to complete discolouration when added to solutions of **2** in THF. This discolouration is accompanied by a splitting of the ^19^F NMR resonances into two distinct sets of C_6_F_5_ resonances, suggesting the formation of a water adduct. Since DFT calculations (MPW1PW91 functional with mixed basis sets: aug-cc-pVTZ for Sb and P, 6-31G for C, 6-31+G(d′) for Cl, F and O) suggest that the HOMO and LUMO are based on the catecholate and antimony moiety respectively (Fig. 2b and c), we propose that the observed discolouration results from a disruption of the LUMO as a result of coordination of a base to the antimony atom. To probe this possibility more carefully, we carried out a spectrophotometric titration of **2** with Et_3_PO (Scheme 1, Fig. 3a).^50,51^ The binding isotherm and the abrupt inflexion at one equivalent unambiguously indicate the coordination of a single molecule of Et_3_PO to the stiborane (Fig. 3a). The formation of adduct Et_3_PO·**2** has been confirmed by X-ray diffraction which shows the presence of two molecules in the asymmetric unit (Fig. 3b). In both molecules, which have very similar structures, the antimony atom adopts an octahedral geometry. The antimony atom is bound to the phosphine oxide with an average Sb–O distance of 2.110(4) Å, which is only slightly longer than the value expected for a typical single bond (∑_CR_(Sb,O) = 2.03 Å).^52^ Surprisingly, this distance is much shorter than the reported distance in the monocation [(Et_3_PO)SbPh_4_]^+^ (2.406(2) Å), and essentially identical to the Sb–O distances in the dication [(Et_3_PO)_2_SbPh_3_]^2+^ (2.089(3) Å),^23^ thus pointing to a considerable Lewis acidic character for **2**. In the ^31^P NMR spectrum, the sharpness and location of the resonance of the Et_3_PO·**2** adduct remained unaffected by the presence of excess Et_3_PO base, in either chloroform (73.5 ppm) or acetonitrile (77.3 ppm). The additional peak, the chemical shift of which is consistent with free Et_3_PO (51.2 ppm in chloroform and 51.0 ppm in acetonitrile), indicates that if exchange occurs, it is very slow on the NMR timescale. This is reminiscent of the behaviour of B(C_6_F_5_)_3_ which also shows two ^31^P NMR signals when an excess of Et_3_PO is present. More importantly, comparison of the ^31^P NMR chemical shift of (Et_3_PO)B(C_6_F_5_)_3_ (76.6 ppm in chloroform and 81.2 ppm in acetonitrile) with those of Et_3_PO·**2** (73.5 ppm in chloroform and 77.3 ppm in acetonitrile) shows that stiborane **2** is a potent Lewis acid, surpassed only marginally by B(C_6_F_5_)_3_. The effect of phenyl group perfluorination in **2** was further assessed by a comparison with the behaviour of the non-fluorinated analogue SbPh_3_(O_2_C_6_Cl_4_).^53^ Only a single ^31^P NMR resonance is observed when more than one equivalent of Et_3_PO is present, implying that (Et_3_PO)SbPh_3_(O_2_C_6_Cl_4_) undergoes rapid exchange with free Et_3_PO. It is also interesting to note that the chemical shift of (Et_3_PO)SbPh_3_(O_2_C_6_Cl_4_), which was measured using a SbPh_3_(O_2_C_6_Cl_4_)/Et_3_PO mixture containing a ten-fold excess of the stiborane, is 62.7 ppm in chloroform. This value is significantly less downfield than that of Et_3_PO·**2** (73.5 ppm) further illustrating the beneficial effects imparted by the presence of perfluorinated phenyl groups.

**Fig. 4:**
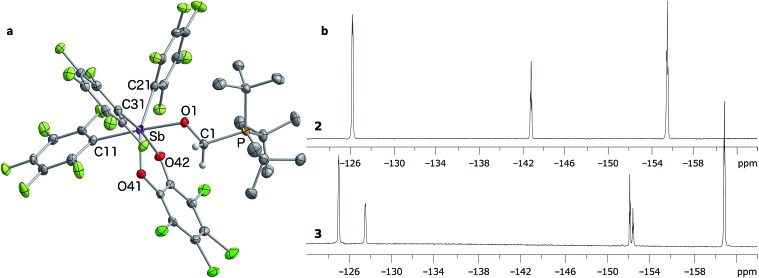
Solid-state structure of **3** (a) with thermal ellipsoids drawn at the 50% probability level. Hydrogen atoms (barring methylene H) and solvent molecules omitted for clarity. Select distances [Å] and angles [°]: Sb–O(1) 2.0384(17), P–C(1) 1.841(3), O(1)–C1 1.397(3), O(1)–Sb–C(11) 177.05(8), Sb–O(1)–C(1) 127.72(15), P–C(1)–O(1) 111.81(17), Sb–O(1)–C(1)–P 163.33(10). The ^19^F NMR spectrum of **3** (bottom b) is shown in comparison to that of stiborane **2** (top b).

Next, we tested the compatibly of this potent Lewis acid with phosphines. Upon mixing with P^*t*^Bu_3_, ^31^P and ^19^F NMR spectroscopy indicates that the two molecules do not form a Lewis adduct. Over time however, ^19^F NMR spectroscopy suggests that stiborane **2** is slowly converted into stibine **1**, implying that P^*t*^Bu_3_ acts as a reducing agent. This observation parallels that made by Burford on the reduction of antimony(v) species by phosphines.^25,54^ Hence, while we see evidence of steric frustration, the pair **2**/P^*t*^Bu_3_ is reactive and thus not chemically frustrated. Nevertheless, this redox reaction is relatively slow such that the pair **2**/P^*t*^Bu_3_ can participate in reactions before the redox process becomes deleterious. While no reaction could be observed with CO_2_, addition of P^*t*^Bu_3_ to a solution of **2** and paraformaldehyde (PFA) in dichloromethane at room temperature leads to the fast disappearance of the yellow colour, indicating consumption of **2**. The product of this reaction has been identified as the formaldehyde-trapping complex **3** (Scheme 1), characterized by a broad ^31^P NMR resonance at 43.0 ppm. The methylene bridge gives rise to a ^1^H NMR resonance at 4.40 ppm and a ^13^C NMR resonance at 38.5 ppm (^1^ *J*_CP_ = 47 Hz). The ^19^F NMR spectrum shows that one of the C_6_F_5_ ligands is not equivalent to the other two (Fig. 4b), in agreement with the existence of an octahedral geometry at antimony. In the solid state, the Sb–O(1)–C(1)–P bridge is almost planar (163.33(10)°), with a C(1)–O(1) distance of 1.397(3) Å that is typical for a C–O single bond (∑_CR_(C,O) = 1.38 Å, Fig. 4a).^52^ This full activation of the double bond is supported by a Sb–O(1) distance of 2.0384(17) Å and a P–C(1) distance of 1.841(3) Å, a set of values close to those expected for Sb–O and P–C single bonds, respectively (∑_CR_(Sb,O) = 2.03 Å, ∑_CR_(P,C) = 1.86 Å).^52^

The above chemistry can be carried out with unpurified solvents. Interestingly, no reaction is observed when P^*t*^Bu_3_ is replaced by PPh_3_, suggesting that the basicity of the phosphine is crucial to the outcome of this reaction. Similarly, when P^*t*^Bu_3_ is paired with SbPh_3_(O_2_C_6_Cl_4_) in dichloromethane, no reaction is observed with PFA at room temperature indicating that the Lewis acidity of the stiborane is equally important.

### Synthesis and structure of ambiphilic phosphino-stiboranes

**Scheme 2:**
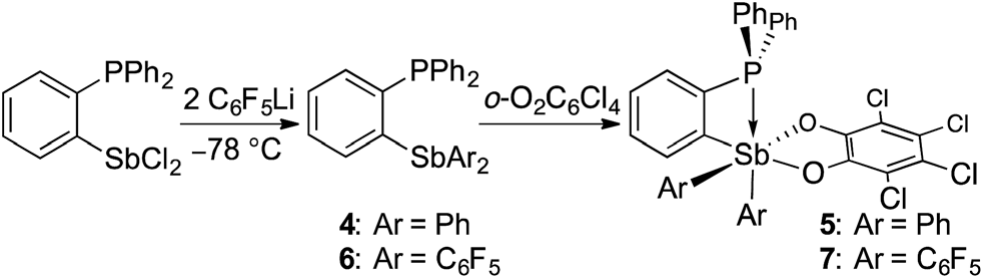
Preparation of ambiphilic phosphino-stiboranes **5** and **7**.

Having confirmed that **2** can participate in FLP reactivity in the presence of a phosphine, we decided to target intramolecular versions of such systems with the phosphine and stiborane units positioned to cooperatively react with incoming substrates. By analogy with ambiphilic *o*-phenylene-bridged phosphinoboranes,^55–57^ we first prepared the yellow stiborane *o*-C_6_H_4_(PPh_2_)(SbPh_2_(O_2_C_6_Cl_4_)) (**5**, 69% yield) by oxidation of the known *o*-C_6_H_4_(PPh_2_)(SbPh_2_)^58^ (**4**) with *o*-chloranil (Scheme 2). Encouraged by the favourable influence of the pentafluorophenyl substituents observed in the case of **2**, we also targeted a fluorinated analog of **5**. To this end, we comproportionated SbCl_3_ and (*o*-(Ph_2_P)C_6_H_4_)_3_Sb at 90 °C to generate *o*-C_6_H_4_(PPh_2_)(SbCl_2_), which was subsequently treated with C_6_F_5_Li in a hexane/diethyl ether solution at –78 °C to afford *o*-C_6_H_4_(PPh_2_)(Sb(C_6_F_5_)_2_) (**6**). After confirming its solid-state structure (see ESI†), stibine **6** (Scheme 2) was treated with *o*-chloranil to afford the deep-orange phosphino-stiborane *o*-C_6_H_4_(PPh_2_)(Sb(C_6_F_5_)_2_(O_2_C_6_Cl_4_)) (**7**, 85% yield). The ^31^P NMR chemical shifts of **5** (25.5 ppm) and **7** (53.0 ppm) are notably downfield from those of PPh_3_ (–6.0 ppm), **4** (–5.1 ppm) and **6** (–8.8 ppm). Such downfield shifts suggest that the phosphorus atom in **5** and **7** interacts with the *ortho*-antimony centre. This interaction appears much stronger than the P→Sn interaction in *o*-C_6_H_4_(PPh_2_)(SnPh_2_Cl) for which a ^31^P NMR chemical shift of –1.0 ppm was measured.^59^

**Fig. 5:**
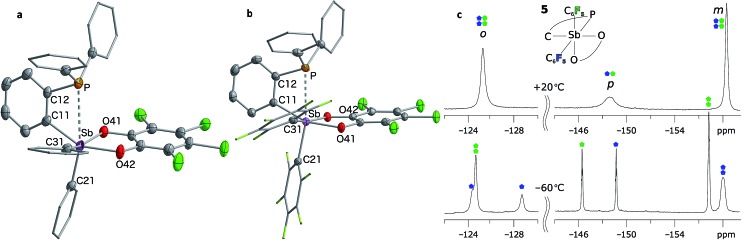
Solid-state structures of phosphino-stiboranes **5** (a) and **7** (b) with thermal ellipsoids drawn at the 50% probability level. Phenyl and C_6_F_5_ groups are drawn in wireframe, while hydrogen atoms and solvent molecules omitted for clarity. Select distances [Å] and angles [°] for **5**: Sb–P 3.0268(12), Sb–C(11) 2.149(3), Sb–C(21) 2.118(4), Sb–C(31) 2.138(3), P–Sb–C(21) 161.70(10), Sb–C(11)–C(12) 115.0(3), P–C(12)–C(11) 112.9(3), C(11)–Sb–O(42) 153.50(13), C(31)–Sb–O(41) 157.15(13), Sb–C(11)–C(12)–P 1.8(3); for **7**: Sb–P 2.8081(9), Sb–C(11) 2.151(2), Sb–C(21) 2.164(3), Sb–C(31) 2.186(3), P–Sb–C(21) 166.46(7), Sb–C(11)–C(12) 109.41(17), P–C(12)–C(11) 111.91(19), C(11)–Sb–O(42) 160.94(8), C(31)–Sb–O(41) 162.30(8), Sb–C(11)–C(12)–P 2.3(2). ^19^F NMR spectrum (c) of stiborane **7** at room temperature (top) resolves into two distinct C_6_F_5_ environments at low temperatures (bottom). The assignments assume that the rotation of the C_6_F_5_ group (represented in blue) *trans* to the phosphine bond is restricted due to greater steric crowding, leading to additional splitting of the resonances.

The presence of the P→Sb interaction in **5** and **7** was confirmed in the solid state (Fig. 5a and b). The presence of these interactions is derived from the short P–Sb distances (**5**: 3.0268(12) Å; **7**: 2.8082(11) Å) as well as from the values of the P–C(12)–C(11) (**5**: 112.9(3)°; **7**: 111.9(2)°) and Sb–C(11)–C(12) angles (**5**: 115.0(3)°; **7**: 109.4(2)°) that are distinctly compressed when compared to the ideal value of 120°. Consistent with the ^31^P NMR data, these structural features indicate that the C_6_F_5_ groups afford a more acidic antimony centre in **7**, and accordingly, a stronger P→Sb interaction. Short P–Sb separations have also been observed in derivatives in which the two moieties are linked by a *peri*-naphthalene linker such as 5-(Ph_2_P)–6-(Cl_2_Sb)–Ace (2.808(1) Å) or 5-(^i^Pr_2_P)–6-(Cl_2_Ph_2_Sb)–Ace (2.9925(8) Å, Ace = acenaphthylene).^60^ Although these P–Sb distances are comparable to those measured in **5** and **7**, it can be expected that the strain imposed by the use of the *ortho*-phenylene backbone will fragilize this linkage, opening the door for reactivity in the P/Sb pocket.

**Fig. 6:**
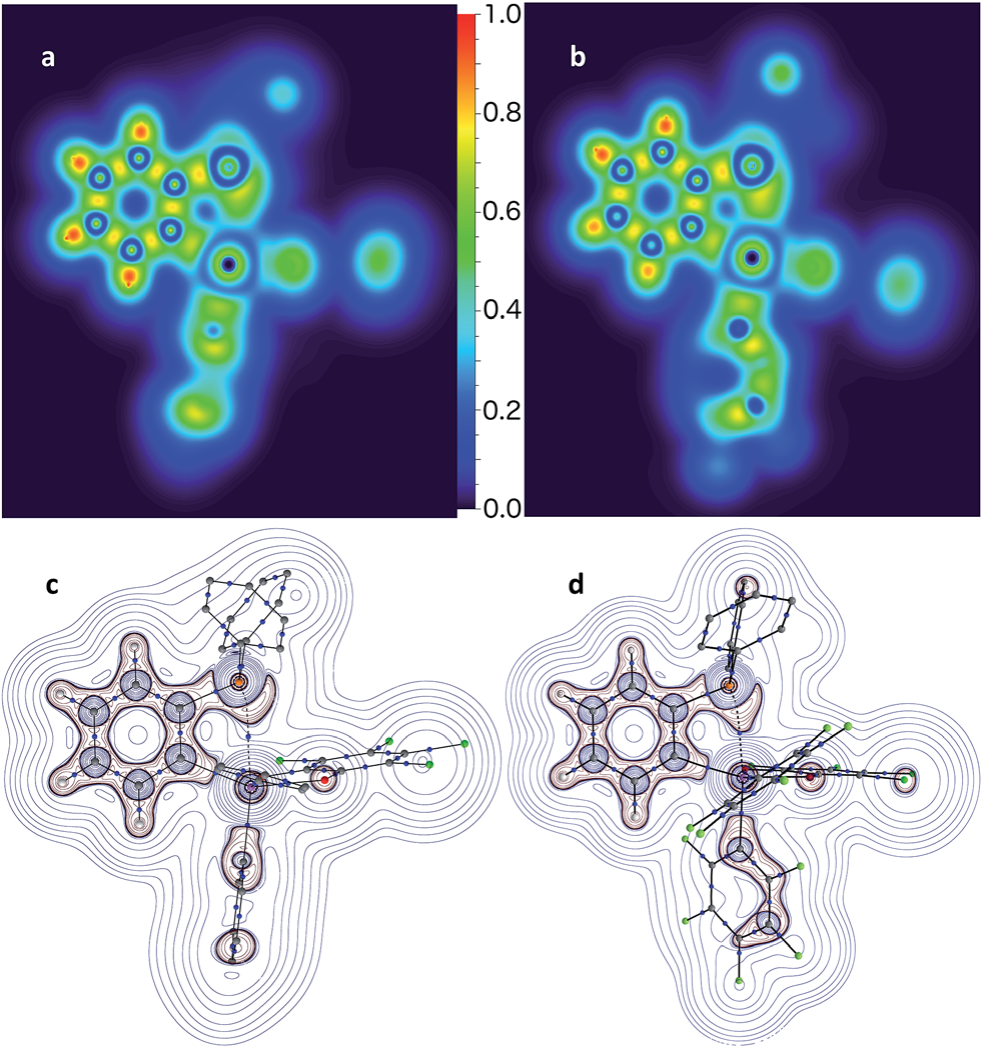
Localized orbital locator maps points for **5** (a) and **7** (b) through the P–C(11)–Sb plane, and corresponding QTAIM bond paths and bond critical points (blue dots) with overlaid contour plots of Laplacian (∇^2^) of *ρ*(*r*) (c and d) through the same plane show skewing of the phosphorus lone-pair towards the antimony atom. Hydrogen atoms and bond paths with *ρ*(*r*) at critical points under 0.01*e* × *r*_Bohr_^–3^ are omitted for clarity.

The P→Sb interactions in **5** and **7** were further analysed computationally using density functional theory methods. Geometry optimisations using the MPW1PW91 functional and mixed basis sets (aug-cc-pVTZ for Sb and P; 6-31+G(d′) for Cl, F and O; 6-31G for C and H) yielded structures that are in good agreement with those experimentally determined. In particular, the calculated P–Sb separations (**5**: 2.9666 Å; **7**: 2.7691 Å) are very close to those measured by X-ray diffraction (**5**: 3.0268(12) Å; **7**: 2.8082(11) Å) and unambiguously show that the phosphorus lone-pair is engaged with the Lewis acidic antimony center. Visualisation of the Localized Orbital Locator (LOL), as defined by Becke and Tsirelson,^61^ reveals a slow electron region oriented towards the acidic antimony atom, with a stronger protrusion in stiborane **7** (Fig. 6a and b). Topological analysis of the electron density (*ρ*) performed according to the atoms in molecules (AIM) method^62^ (Fig. 6c and d) reveals an increased electron density at the P–Sb bond critical point (*ρ*(BCP): 0.054 *e* × *r*_Bohr_^–3^ for **7** and 0.035 *e* × *r*_Bohr_^–3^ for **5**) and a larger delocalisation index (*δ*(Sb,P): 0.38 for **7** and 0.24 for **5**) in the case of **7**. These differences confirm the stronger P→Sb interaction present in stiborane **7**. Moreover, the decrease in the value of the Laplacian (∇^2^) of *ρ* at the BCP (from 0.027 *e* × *r*_Bohr_^–5^ in **5** to 0.008 *e* × *r*_Bohr_^–5^ in **7**, respectively) is suggestive of a decreased donor–acceptor character and an increased covalent character for the P–Sb bond of **7**.^63^ It follows that the two C_6_F_5_ rings present in **7** generate a more acidic antimony centre and a stronger P→Sb interaction. The strength of this interaction is also reflected by the appearance of two distinct C_6_F_5_ environments in the low temperature ^19^F NMR spectrum of **7** (Fig. 5c). A line-shape analysis indicates that this fluxional process has an activation barrier Δ*H* ^‡^ of 10.2 kcal mol^–1^ (see ESI†), a value possibly correlated to the strength of the P→Sb interaction.

The selective addition of *o*-chloranil to the antimony rather than to the phosphorus atom in **4** and **6**, together with the lack of any observed redox isomerisation involving products **5** and **7**, are quite surprising considering the existing literature on phosphine oxidation by antimony(v) compounds.^25,54^ It is likely that the stabilisation afforded by the donation of the phosphorus lone-pair to the neighbouring antimony atom plays a large role in this selectivity. This stabilisation is also likely responsible for the stability displayed by these two compounds. Indeed, when layered with water, solutions of **5** and **7** in chloroform show no signs of decomposition even after 3 hours at room temperature. Next, we decided to investigate whether the strain imparted by the *o*-phenylene linker could be exploited as a way to induce reactivity in the P/Sb pocket.

### Reaction of ambiphilic phosphino-stiboranes with formaldehyde

**Scheme 3:**
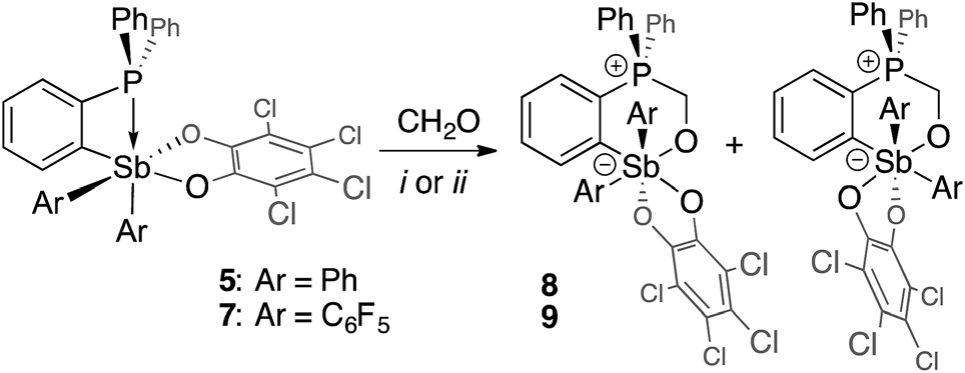
Insertion of a formaldehyde unit into the Sb–P pockets of **5** and **7**: (i) (CH_2_O)_*n*_ in toluene at 70 °C; (ii) aqueous CH_2_O at room temperature.

**Fig. 7:**
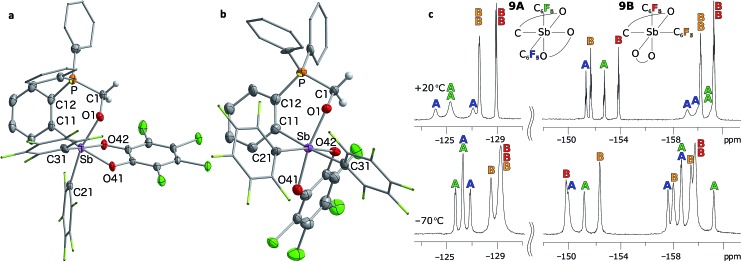
Solid-state structures of the two isomers of **9**, namely **9A** (a) and **9B** (b), with thermal ellipsoids drawn at the 50% probability level. The C_6_F_5_ groups are drawn in wireframe while the hydrogen atoms (barring methylene H) and solvent molecules are omitted for clarity. Select distances [Å] and angles [°] for **9A**: Sb–O(1) 2.038(2), P–C(1) 1.813(3), O(1)–C(1) 1.391(4), Sb–P 3.6526(11), O(1)–Sb–C(21) 166.32(10), C(11)–Sb–O(41) 171.07(10), Sb–O(1)–C(1)–P 73.3(3); for **9B**: Sb–O(1) 2.003(8), P–C(1) 1.798(12), O(1)–C(1) 1.409(15), Sb–P 3.697(3), O(1)–Sb–O(41) 167.9(3), C(11)–Sb–C(31) 174.9(4), Sb–O(1)–C(1)–P 79.9(9). The ^19^F NMR spectra of **9** (c) show resolved resonances at low temperatures (see ESI†). The assignments shown assume that the rotation of the C_6_F_5_ groups (represented in blue for isomer **9A** and in orange for isomer **9B**) are restricted due to greater steric crowding, leading to additional splitting of the resonances.

**Fig. 8:**
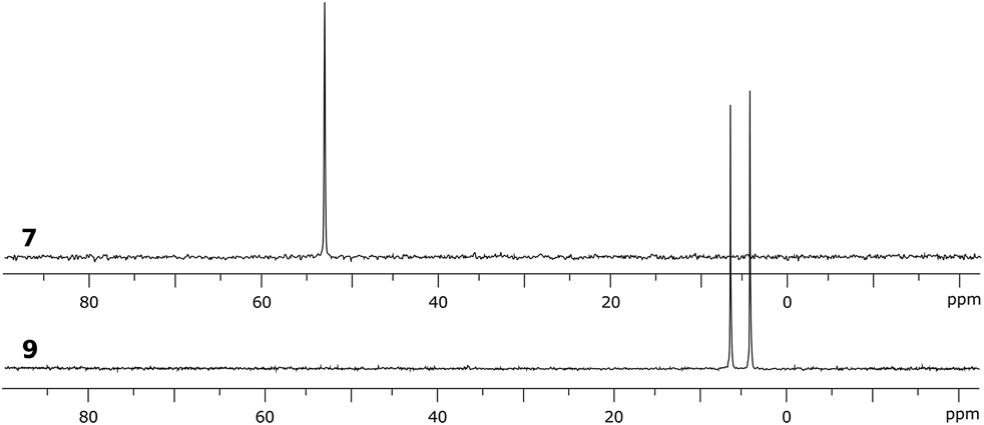
^31^P NMR spectra of phosphino-stiborane **7** and its CH_2_O-insertion product **9** containing two isomers.

Although no reaction is observed at room temperature, heating mixtures of stiboranes **5** or **7** and PFA to 70 °C in toluene resulted in the formation of the corresponding formaldehyde-insertion products **8** and **9**, respectively (Scheme 3). Formation of compounds **8** and **9**, which have been characterized by conventional means including elemental analysis, indicate that P→Sb bond of **5** or **7** can indeed be activated thereby unmasking the Lewis acid and Lewis basic sites of these derivatives. Multinuclear NMR spectroscopy suggests that **8** and **9** exist as pairs of isomers as supported by the detection of two ^31^P NMR resonances at 3.0 ppm and 4.3 ppm for **8**, and at 4.0 ppm and 6.3 ppm for **9** (Fig. 8). In accordance with the existence of two isomers, two distinct methylene groups are observed in the ^1^H NMR spectra of each product mixture. In addition, each isomer of **9** possesses two distinct C_6_F_5_ environments as seen in the ^19^F NMR spectrum (Fig. 7c). The ^19^F NMR spectrum is further complicated by the hindered rotation of the C_6_F_5_ substituents about the Sb–C_*ipso*_ bonds, leading to 20 resonances at –70 °C, some of which show accidental overlap (Fig. 7c). In the case of **9**, we also succeeded in obtaining single crystals of both isomers which are referred to as **9A** and **9B** (Fig. 7a and b). These two isomers only differ in the arrangement of the substituents about the antimony center. In the case of **8**, we only succeeded in crystallizing one isomer, the structure of which is essentially identical to that of **9A** (see ESI†). The length of the C(1)–O(1) (**8**: 1.393(5) Å; **9A**: 1.391(4) Å; **9B**: 1.409(15) Å), Sb–O(1) (**8**: 2.044(3) Å; **9A**: 2.038(2) Å; **9B**: 2.003(8) Å) and P–C(1) bonds (**8**: 1.800(5) Å; **9A**: 1.813(3) Å; **9B**: 1.798(12) Å) are very similar to those observed for ^*t*^Bu_3_P–CH_2_O–Sb(C_6_F_5_)_3_(O_2_C_6_Cl_4_) (**3**) indicating complete activation of the formaldehyde monomer. The only notable difference is the value of the Sb–O(1)–C(1)–P dihedral angles which are constrained to much smaller values in the cyclic *ortho*-phenylene systems (**8**: 73.8(4)°; **9A**: 73.3(3)°; **9B**: 79.9(9)°) than in **3** (163.33(10)°).

**Fig. 9:**
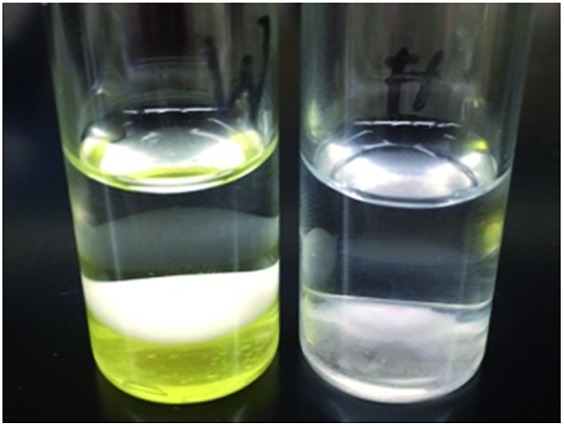
Dichloromethane solutions of phosphino-stiborane **7** layered with water (left) and 0.1% aqueous formaldehyde (right) after sonication for 90 min.

Given the stability of these species to water and their reactivity towards formaldehyde, we decided to test whether such systems could be used for the colourimetric detection of aqueous formaldehyde. This study was further motivated by the knowledge that formaldehyde is carcinogenic and widely used in industry.^64^ Given its higher solubility in dichloromethane, the phosphino-stiborane **7** was selected for these studies. The feasibility of this approach was first tested using a commercial formaldehyde aqueous solution (37 wt%, 12 M, 0.5 mL) layered with a toluene solution of **7** (10 mM, 0.5 mL). At this concentration, the biphasic reaction is fast, necessitating only 5 min of vigorous shaking for complete conversion of **7** into **9**, the formation of which was confirmed by NMR spectroscopy. With the view of simulating conditions that would approach those of environmental samples, we also tested more dilute conditions. Layering of a solution of **7** in dichloromethane (10 mM, 0.5 mL) with an aqueous solution of containing formaldehyde (35 mM, 2.5 mL, 18 equiv.) and the neutral surfactant Triton X-100 (0.045 M), led to the progressive disappearance of the yellow colouration upon sonication. The yellow colouration was no longer apparent after 90 minutes (Fig. 9), as consumption of **7** and formation of the two isomers of **9** was confirmed by ^31^P and ^19^F NMR spectroscopy. By contrast, the yellow colouration persisted even after 2 weeks when the aqueous layer contained the surfactant Triton X-100 without any formaldehyde. These results show that **7** can be used for the molecular recognition and colourimetric detection of formaldehyde in aqueous solutions.

## Conclusions

We conclude this paper by making two separate points. The first one relates to the synthesis and properties of the stiborane **2**, a new fluorinated main group reagent which approaches the Lewis acidity of B(C_6_F_5_)_3_ while displaying a remarkable tolerance to moisture when in the solid state or when dissolved in non-polar solvents such as chloroform. The potency of this new Lewis acid, which is illustrated by the isolation of Et_3_PO adduct as well as its use in combination with P^*t*^Bu_3_ for the complexation of formaldehyde, suggests a broad range of applications, several of which are currently being investigated in our laboratory. The second point pertains to the demonstration that fluorinated stiborane units can also be decorated by pendent phosphines to generate ambiphilic phosphino-stiborane derivatives as in the case of **7**. This compound is stabilized by formation of an intramolecular P→Sb interaction, which makes it remarkably inert to water. Despite this apparent stability, the phosphino-stiborane **7** reacts swiftly with formaldehyde to afford the corresponding addition compound **9**. This unique reactivity, which is accompanied by a colourimetric turn-off response, can be implemented using dilute aqueous formaldehyde solutions thereby demonstrating the potential that this frustrated Lewis pair chemistry holds in the domain of molecular recognition and sensing.^65^

## Experimental

### General synthetic procedures

*Solutions of C*_*6*_ *F*_*5*_ *Li should be kept cold at all times to prevent explosions!* Manipulations involving phosphines were performed under an inert atmosphere of purified N_2_ using Schlenk line or glovebox techniques with anhydrous, oxygen-free solvents. All other manipulations, including aqueous formaldehyde tests, were performed under atmospheric conditions using unpurified solvents. ^1^H and ^13^C NMR spectra were obtained on a Varian Unity Inova 400 FT NMR instrument and were referenced to residual CDCl_3_ solvent signals (^1^H at 7.26 ppm, ^13^C at 77.16 ppm). ^31^P and ^19^F NMR spectra were referenced externally to 85% H_3_PO_4_ (0.0 ppm) and BF_3_·OEt_2_ (–153.0 ppm), respectively. UV-Vis spectra were recorded on a Shimadzu UV-2501 spectrometer. Elemental analysis determinations were performed by Atlantic Microlab, Inc., Norcross, GA. SbPh_3_(O_2_C_6_Cl_4_),^53^ *o*-C_6_H_4_(PPh_2_)(SbPh_2_),^58^ and *o*-C_6_H_4_(PPh_2_)_3_Sb^66^ were prepared according to the literature protocols, while the other reagents were purchased from commercial sources. NMR fits were done with the gNMR^67^ software package.

#### Synthesis of stiborane **2**

Solid *o*-chloranil (960 mg, 3.90 mmol, 1.0 equiv.) was added to a dichloromethane (5 mL) solution of Sb(C_6_F_5_)_3_ (2.43 g, 3.90 mmol, 1.0 equiv.). The resulting red suspension was stirred for 10 min, after which the resulting precipitate was collected, washed with pentane, and dried, yielding an orange powder consisting of analytically pure **2**(CH_2_Cl_2_) (2.57 g, 2.69 mmol, 69% yield). X-ray quality crystals of **2**(CHCl_3_) were obtained by layering a chloroform solution with pentane. ^13^C{^1^H} NMR (CDCl_3_, 20 °C, 100 MHz) *δ*: 147.0 (br d, ^1^ *J*_CF_ = 250 Hz, *o*-C_6_F_5_), 145.0 (dt, ^1^ *J*_CF_ = 260 Hz, ^3^ *J*_CF_ = 13 Hz, *p*-C_6_F_5_), 142.5 (*C*O), 138.2 (dt, ^1^ *J*_CF_ = 260 Hz, ^3^ *J*_CF_ = 17 Hz, *m*-C_6_F_5_), 123.5 (*C*Cl), 117.6 (*C*Cl), ≈112 (br, *C*Sb) ppm. ^19^F NMR (CDCl_3_, 20 °C, 376 MHz) *δ*: –125.6 (d, ^3^ *J*_FF_ = 19 Hz, 6F, *o*), –147.8 (t, ^3^ *J*_FF_ = 20 Hz, 3F, *p*), –158.1 (t, ^3^ *J*_FF_ = 19 Hz, 6F, *m*) ppm. Elemental analysis found (calcd for C_25_H_2_Cl_6_O_2_F_15_Sb) [%]: C 31.69 (31.48), H 0.12 (0.21).

#### Et_3_PO adduct of **2**

^1^H NMR (CDCl_3_, 20 °C, 400 MHz) *δ*: 1.47 (br, 6H, *C*H_2_), 0.88 (br, 9H, *C*H_3_) ppm. ^19^F NMR (CDCl_3_, 20 °C, 376 MHz) *δ*: –128.6 (br, *W*_1/2_ = 50 Hz, 4F, *o* ^*cis*^), –129.5 (br, *W*_1/2_ = 50 Hz, 2F, *o* ^*trans*^), –152.4 (t, ^3^ *J*_FF_ = 20 Hz, 2F, *p* ^*cis*^), –152.8 (t, ^3^ *J*_FF_ = 20 Hz, 1F, *p* ^*trans*^), –162.6 (t, ^3^ *J*_FF_ = 20 Hz, 4F, *m* ^*cis*^), –162.7 (t, ^3^ *J*_FF_ = 20 Hz, 2F, *m* ^*trans*^) ppm. ^31^P{^1^H} NMR (CDCl_3_, 20 °C, 162 MHz) *δ*: 73.5 (s) ppm.

#### Synthesis of compound **3**

A dichloromethane (3 mL) solution of P^*t*^Bu_3_ (85 mg, 0.42 mmol, 1.0 equiv.) was added dropwise to an orange dichloromethane (4 mL) suspension containing **2**(CH_2_Cl_2_) (420 mg, 0.44 mmol, 1.0 equiv.) and paraformaldehyde (50 mg, 1.66 mmol, 4 equiv.). The resulting mixture turned pale yellow within 5 min, and was further stirred for 2 h, after which it was filtered through a pad of Celite to remove the excess paraformaldehyde. The filtrate was layered with hexane, and the resulting pale yellow crystals were collected, washed and dried to yield pure **3** (353 mg, 0.30 mmol, 72% yield). ^1^H NMR (CDCl_3_, 20 °C, 400 MHz) *δ*: 4.40 (d, ^2^ *J*_HP_ = 1.7 Hz, 2H, *C*H_2_), 1.36 (d, ^4^ *J*_HP_ = 13.6 Hz, 27H, *C*H_3_) ppm. ^13^C{^1^H} NMR (CDCl_3_, 20 °C, 100 MHz) *δ*: 148 (br d, ^1^ *J*_CF_ = 250 Hz, *o*-C_6_F_5_), 146.5 (*C*O), 142 (br d, ^1^ *J*_CF_ = 250 Hz, *p*-C_6_F_5_), 137 (br d, ^1^ *J*_CF_ = 250 Hz, *m*-C_6_F_5_), 123 (vbr s, *C*Sb), 119.0 (*C*Cl), 116.0 (*C*Cl), ppm, 56.1 (d, ^1^ *J*_CP_ = 47 Hz, P*C*H_2_), 38.5 (d, ^1^ *J*_CP_ = 26 Hz, *C*(CH_3_)_3_), 29.3 (*C*H_3_) ppm. ^19^F NMR (CDCl_3_, 20 °C, 376 MHz) *δ*: –125.0 (d, ^3^ *J*_FF_ = 13 Hz, 4F, *o* ^*cis*^), –127.5 (d, ^3^ *J*_FF_ = 13 Hz, 2F, *o* ^*trans*^), –152.1 (t, ^3^ *J*_FF_ = 20 Hz, 2F, *p* ^*cis*^), –152.4 (t, ^3^ *J*_FF_ = 20 Hz, 1F, *p* ^*trans*^), –160.1 (m, 6F, *m*) ppm. ^31^P{^1^H} NMR (CDCl_3_, 20 °C, 162 MHz) *δ*: 42.7 (br s, *W*_1/2_ = 20 Hz) ppm. Elemental analysis found (calcd for C_37_H_29_Cl_4_F_15_O_3_PSb) [%]: C 40.56 (40.36), H 2.50 (2.65).

#### Synthesis of stiborane **5**

A red solution of *o*-O_2_C_6_Cl_4_ (382 mg, 1.55 mmol, 1.0 equiv.) in acetone (10 mL) was added to a stirring suspension of stibine *o*-C_6_H_4_(PPh_2_)(SbPh_2_) (834 mg, 1.55 mmol, 1.0 equiv.) in acetone (10 mL). The resulting mixture was stirred for 2 min until it became a clear, yellow solution, at which point it was stored at –20 °C for 12 h. The resulting precipitate was collected on a fritted filter, washed with pentane, and dried under reduced pressure to yield crystalline, bright-yellow solids consisting of analytically pure stiborane **5** over two batches (941 mg, 1.20 mmol, 77% yield). ^1^H NMR (CDCl_3_, 20 °C, 400 MHz) *δ*: 7.74 (d, 4H, ^2^ *J*_HH_ = 6.8 Hz, *o*-Ph^Sb^), 7.52–7.58 (m, 2H, C_6_H_2_ *H*_2_), 7.38–7.49 (m, 8H, C_6_ *H*_2_H_2_, *m*-Ph^Sb^, *p*-Ph^Sb^), 7.25–7.30 (m, 2H, *p*-Ph^P^), 7.15 (t, 4H, ^3^ *J*_HH_ = 7.3 Hz, *m*-Ph^P^), 6.90 (pseudo t, 4H, ^3^ *J*_HH_ ≈ ^3^ *J*_HP_ = 8.4 Hz, *o*-Ph^P^) ppm. ^13^C{^1^H} NMR (CDCl_3_, 20 °C, 100 MHz) *δ*: 160.4 (d, ^1^ *J*_CP_ = 98 Hz, 1-C_6_HCP4), 145.1 (*C*O), 139.2 (d, ^2^ *J*_CP_ = 19 Hz, 6-C_6_H_4_), 135.9 (d, ^3^ *J*_CP_ ≈ 1 Hz, 3/5-C_6_H_4_), 134.8 (*o*-Ph^Sb^), 132.7 (*i*-Ph^Sb^), 132.6 (d, ^2^ *J*_CP_ = 15 Hz, *o*-Ph^P^), 132.5 (d, ^2^ *J*_CP_ = 28 Hz, *i*-Ph^P^), 132.4 (d, ^2^ *J*_CP_ = 5 Hz, Sb*C* ^C_6_H_4_^), 131.7 (d, ^3^ *J*_CP_ = 3 Hz, 5/3-C_6_H_4_), 131.3 (4-C_6_H_4_), 131.2 (*p*-Ph^Sb^), 129.7 (d, ^4^ *J*_CP_ ≈ 1 Hz, *p*-Ph^P^), 129.6 (*m*-Ph^Sb^),128.6 (d, ^3^ *J*_CP_ = 9 Hz, *m*-Ph^P^), 119.9 (*C*Cl), 116.9 (*C*Cl) ppm. ^31^P{^1^H} NMR (CDCl_3_, 20 °C, 162 MHz) *δ*: +25.5 ppm. Elemental analysis found (calcd for C_36_H_24_Cl_4_O_2_PSb) [%]: C 55.30 (55.21), H 3.28 (3.09).

#### Synthesis of stibine **6**

Neat SbCl_3_ (173 mg, 0.76 mmol, 0.67 equiv.) and (*o*-C_6_H_4_(PPh_2_))_3_Sb (347 mg, 0.38 mmol, 0.33 equiv.) were allowed to react at 90 °C for 36 h to afford *o*-C_6_H_4_(PPh_2_)(SbCl_2_) as crude product. This was dissolved in 1 : 1 Et_2_O/THF (volume) and the resulting solution was slowly added to a cooled (–78 °C) solution of C_6_F_5_Li. The latter was prepared fresh by addition of and a 2.2 M solution of ^*n*^BuLi in hexanes (1.1 mL, 2.42 mmol, 2.2 equiv.) to a diethyl ether (30 mL) solution of C_6_F_5_Br (600 mg, 2.43 mmol, 2.2 equiv.) and stirring for 1 h at –78 °C. The final combined mixture was allowed to warm up to room temperature under stirring, as a white precipitate started to appear. After 16 h, the volatiles were removed and the off-white was washed with dichloromethane through a pad of Celite to remove the LiCl by-product. Volatiles from the filtrate were removed again, and the white solid was washed with pentane to yield pure stibine **6** as a white powder (451 mg, 0.63 mmol, 52% yield). ^1^H NMR (CDCl_3_, 20 °C, 400 MHz) *δ*: 7.58 (br s, *W*_1/2_ = 13 Hz, 1H, 3-C_6_H_3_ *H*), 7.40–7.45 (m, 2H, C_6_H_2_ *H*_2_), 7.35–7.26 (m, 6H, *o*-Ph, *p*-Ph), 7.22 (pseudo qr, *J* = 4.4 Hz, 1H, C_6_H_3_ *H*), 7.11 (td, ^3^ *J*_HH_ = 8.2 Hz, ^4^ *J*_HP_ = 1.3 Hz, 4H, *m*-Ph) ppm. ^13^C{^1^H} NMR (CDCl_3_, 20 °C, 100 MHz) *δ*: 148.0 (br d, ^1^ *J*_CF_ = 240 Hz, *o*-C_6_F_5_), 145.1 (d, ^1^ *J*_CP_ = 54 Hz, 1-C_6_H_4_), 142.4 (d, ^3^ *J*_CP_ = 4.2 Hz, 3-C_6_H_4_), 142.1 (br d, ^1^ *J*_CF_ = 254 Hz, *p*-C_6_F_5_), 137.1 (br d, ^1^ *J*_CF_ = 260 Hz, *m*-C_6_F_5_), 135.9 (d, ^2^ *J*_CP_ = 16.6 Hz, 6-C_6_H_4_), 135.3 (s, 4-C_6_H_4_), 134.5 (d, ^3^ *J*_CP_ = 3.8 Hz, 5-C_6_H_4_), 132.9 (d, ^2^ *J*_CP_ = 17.5 Hz, *m*-Ph), 130.7 (d, ^1^ *J*_CP_ = 57 Hz, *i*-Ph), 129.2 (s, *p*-Ph), ∼129 (br s, *i*-C_6_F_5_), 128.7 (d, ^3^ *J*_CP_ = 7.4 Hz, *m*-Ph), 108.4 (br m, 2-C_6_H_4_) ppm. ^19^F NMR (CDCl_3_, 20 °C, 376 MHz) *δ*: –120.6 (d, ^3^ *J*_FF_ = 21.7 Hz, 4F, *o*-C_6_F_5_), –150.5 (t, ^3^ *J*_FF_ = 19.9 Hz, 2F, *p*-C_6_F_5_), –159.3 (pseudo t, 4F, *m*-C_6_F_5_) ppm. ^31^P{^1^H} NMR (CDCl_3_, 20 °C, 162 MHz) *δ*: –8.8 ppm. Elemental analysis found (calcd for C_61_H_30_F_20_Cl_2_P_2_Sb_2_) [%]: C 48.67 (48.23), H 2.13 (1.99).

#### Synthesis of stiborane **7**

Solid *o*-chloranil (265 mg, 1.08 mmol, 1.0 equiv.) was added to a stirring suspension of stibine **6** (780 mg, 1.08 mmol, 1.0 equiv.) in dichloromethane (4 mL). The resulting mixture was stirred for 2 min until it became a clear, orange solution, at which point it was filtered through a cotton plug and layered with hexane (3 mL). After a day, the liquid was decanted and the resulting orange crystals were washed with hexane and dried under reduced pressure to yield a bright-orange, crystalline solid consisting of analytically pure **7** (812 mg, 0.62 mmol, 57% yield). ^1^H NMR (CDCl_3_, 20 °C, 400 MHz) *δ*: 7.94 (br d, ^3^ *J*_HH_ = 7.8 Hz, 1H, 3-C_6_H_4_), 7.82 (tdd, ^3^ *J*_HH_ = 7.5 Hz, ^4^ *J*_HP_ = 5.1 Hz, ^4^ *J*_HH_ = 1.3 Hz, 1H, 5-C_6_H_4_), 7.75 (tdd, ^3^ *J*_HH_ = 7.4 Hz, ^5^ *J*_HP_ = 2.4 Hz, ^4^ *J*_HH_ = 1.2 Hz, 1H, 4-C_6_H_4_), 7.61 (ddd, ^3^ *J*_HH_ = 7.4 Hz, ^3^ *J*_HP_ = 4.5, ^4^ *J*_HH_ = 0.8 Hz, 1H, 6-C_6_H_4_), 7.45 (tq, ^3^ *J*_HH_ = 7.5 Hz, ^4^ *J*_HH_ ≈ ^5^ *J*_HP_ ≈ 1.8 Hz, 2H, *p*-Ph), 7.30 (td, ^3^ *J*_HH_ = 7.8 Hz, ^4^ *J*_HP_ = 2.0 Hz, 4H, *m*-Ph), 7.1 (br s, *W*_1/2_ = 25 Hz, 4H, *o*-Ph) ppm. ^13^C{^1^H} NMR (CDCl_3_, 20 °C, 100 MHz) *δ*: 169.0 (d, ^1^ *J*_CP_ = 115 Hz, 1-C_6_H_4_), 147.0 (br d, ^1^ *J*_CF_ = 247 Hz, *o*-C_6_F_5_), 144.4 (*C*O), 143.1 (br d, ^1^ *J*_CF_ = 260 Hz, *p*-C_6_F_5_), 137.0 (br d, ^1^ *J*_CF_ = 260 Hz, *m*-C_6_F_5_), 134.2, 134.1, 133.4, 133.2, 133.2, 133.0, 132.0, 131.9, 131.5, 131.4, 129.2, 129.1, 125.5 (br d, ^2^ *J*_CP_ = 21 Hz, 2-C_6_H_4_), 121.2 (*C*Cl), 117.5 (*C*Cl), ≈114 (vbr, *i*-C_6_F_5_) ppm. ^19^F NMR (CDCl_3_, 20 °C, 376 MHz) *δ*: –125.1 (br s, *W*_1/2_ = 150 Hz, 4F, *o*-C_6_F_5_), –148.5 (br s, *W*_1/2_ = 400 Hz, 2F, *p*-C_6_F_5_), –158.3 (br s, *W*_1/2_ = 85 Hz, 4F, *m*-C_6_F_5_) ppm. ^31^P{^1^H} NMR (CDCl_3_, 20 °C, 162 MHz) *δ*: +53.0 ppm. Elemental analysis found (calcd for C_36_H_14_Cl_4_F_10_O_2_PSb) [%]: C 44.87 (44.90), H 1.40 (1.47).

#### Synthesis of compound **8**

A yellow suspension of stiborane **5** (250 mg, 0.32 mmol, 1.0 equiv.) and (CH_2_O)_*n*_ (20 mg, 0.66 mmol, 2.0 equiv.) in toluene (5 mL) was placed in a bath at 80 °C and stirred for 4 h, resulting in a pale yellow solution and off-white solids. Volatiles were removed under vacuum, and the solid residue was passed through a layer of Celite with dichloromethane (100 mL) to remove excess polymer. The pale yellow filtrate was concentrated (to 7 mL) and was layered with hexane (7 mL). After a day, the resulting crystalline solid was collected, washed with pentane and dried to yield pure material consisting of colourless compound **8** (197 mg, 0.24 mmol, 76% yield). ^1^H NMR (CDCl_3_, 20 °C, 400 MHz) *δ*: 7.8–7.1 (br m, 24H, C_6_ *H*_5_ and C_6_ *H*_4_), 6.04 (dd, ^2^ *J*_HH_ = 14 Hz, ^2^ *J*_HP_ = 5.6 Hz, C*H*H^major^), 5.35 (dd, ^2^ *J*_HH_ = 14 Hz, ^2^ *J*_HP_ = 3.0 Hz, C*H*H^minor^), 5.00 (dd, ^2^ *J*_HH_ = 14 Hz, ^2^ *J*_HP_ = 1.2 Hz, CH*H* ^major^), 4.88 (dd, CH*H* ^minor^) ppm. ^31^P{^1^H} NMR (CDCl_3_, 20 °C, 162 MHz) *δ*: +4.3 (minor isomer, ≈10%), +3.0 (major isomer, ≈90%) ppm. Compound is not soluble enough for ^13^C{^1^H} NMR analysis. Elemental analysis found (calcd for C_37_H_26_Cl_4_O_3_PSb) [%]: C 54.87 (54.65), H 3.21 (3.22).

#### Synthesis of complex **9**

An orange suspension of stiborane **7** (281 mg, 0.29 mmol, 1.0 equiv.) and (CH_2_O)_*n*_ (20 mg, 0.66 mmol, 2.0 equiv.) in toluene (5 mL) was placed in a bath at 70–80 °C and stirred for 3 h, resulting in a colourless solution. Volatiles were removed under vacuum, and the solid residue was passed with dichloromethane (5 mL) through a glass paper plug to remove excess polymer. The filtrate was layered with hexane (7 mL) and the resulting solid was collected on a fritted glass filter, washed with hexane, and dried under reduced pressure, yielding a white powder consisting of analytically pure compound **9**(CH_2_Cl_2_)_1.5_ (240 mg, 0.22 mmol, 76% yield). Both isomers form in a ≈50 : 50 ratio, but in solution, one isomer slowly converts into the other one; spectra were recorded at a ≈65 : 35 ratio between the two isomers, with **9B** tentatively assigned as the major isomer, and **9A** as the minor. ^1^H NMR (CDCl_3_, 20 °C, 400 MHz) *δ*: 8.07 (br t, 1H^minor^), 8.01 (dd, 1H^major^), 7.85–7.72 (m, ≈4H), 7.69–7.45 (m, ≈9H), 7.38 (t, *J* = 7.36 Hz, 1H^major^), 7.28 (dd, 13 Hz, 7.7 Hz, 1H^major^), 6.91 (dd, ^2^ *J*_HH_ = 15 Hz, ^2^ *J*_HP_ = 4.8 Hz, C*H*H^major^), 5.95 (dd, ^2^ *J*_HH_ = 15 Hz, ^2^ *J*_HP_ = 5.8 Hz, C*H*H^minor^), 5.47 (dd, ^2^ *J*_HH_ = 15 Hz, ^2^ *J*_HP_ = 3.8 Hz, CH*H* ^major^), 5.11 (dd, ^2^ *J*_HH_ = 15 Hz, ^2^ *J*_HP_ = 2.6 Hz, CH*H* ^minor^) ppm. ^13^C{^1^H} NMR (CDCl_3_, 40 °C, 100 MHz) *δ*: 160.2, 160.0, 153 (vbr d, ^1^ *J*_CF_ ≈ 250 Hz), 147.5 (br d, ^1^ *J*_CF_ = 245 Hz), 147.0, 145.8, 145.5, 142 (vbr d, ^1^ *J*_CF_ ≈ 250 Hz), 137.2 (br d, ^1^ *J*_CF_ = 255 Hz), 136.6, 136.5, 135.6, 135.5, 135.3, 135.2, 135.1, 135.0, 135.0, 135.0, 134.9, 134.8, 134.7, 133.8, 133.7, 133.7, 133.6, 133.6, 133.5, 130.7, 130.6, 130.6, 130.5, 130.4, 130.1, 130.0, 129.9, 129.6, 129.5, 125.9, 125.0, 123.2, 122.3, 120.6, 120.5, 120.3, 119.8, 119.5, 119.2, 119.0, 118.7, 118.4, 118.1, 117.8, 117.5, 117.3, 116.4, 63.4 (d, ^1^ *J*_CP_ = 56 Hz, P*C*Hmajor2), 63.1 (d, ^1^ *J*_CP_ = 56 Hz, P*C*Hminor2) ppm. ^19^F NMR (CDCl_3_, 20 °C, 376 MHz) *δ*: –123.1 (br, 1F, *o* ^minor^), –124.2 (br, 2F, *o* ^minor^), –126.0 (br, 1F, *o* ^minor^), –126.5 (d, 2F, ^3^ *J*_FF_ = 19 Hz, *o* ^major^), –127.8 (d, 2F, ^3^ *J*_FF_ = 21 Hz, *o* ^major^), –151.3 (t, 1F, ^3^ *J*_FF_ = 21 Hz, *p* ^minor^), –151.7 (t, 1F, ^3^ *J*_FF_ = 20 Hz, *p* ^major^), –152.8 (t, 1F, ^3^ *J*_FF_ = 20 Hz, *p* ^minor^), –153.9 (t, 1F, ^3^ *J*_FF_ = 21 Hz, *p* ^major^), –159.2 (br, 1F, *m* ^minor^), –160.0 (br, 1F, *m* ^minor^), –160.2 (pseudo t, ^3^ *J*_FF_ = 18 Hz, 2F, *m* ^major^), ≈–161.4 (br, 2F, *m* ^minor^), –161.3 (pseudo t, ^3^ *J*_FF_ = 19 Hz, 2F, *m* ^major^) ppm. ^31^P{^1^H} NMR (CDCl_3_, 20 °C, 162 MHz) *δ*: +6.3 (minor), +4.0 (major) ppm. Elemental analysis found (calcd for C_77_H_38_Cl_14_F_20_O_6_P_2_Sb_2_) [%]: C 41.30 (41.27), H 1.67 (1.71).

#### Biphasic formaldehyde test with stiborane **7**

Two samples containing solutions of stiborane **7** (0.5 mL) from a dichloromethane stock solution (10 mM) in two vials: one vial was layered with (a) an aqueous solution of 0.1 wt% formaldehyde solution (2.5 mL, 18 equiv.) containing Triton X-100 (0.045 M) and (b) a sample of water (3 mL) containing only surfactant Triton X-100 (0.045 M), respectively. The two samples were mixed vigorously in a sonicator until the colouration of the organic layer from the formaldehyde vial (a) disappeared (90 min, displayed in Fig. 9). The organic layers from both samples were analysed by ^19^F and ^31^P NMR spectroscopy.

### Crystallographic details

Diffraction-quality crystals were obtained by layering chloroform, dichloromethane or acetone solutions with hexane and allowing the mixtures to sit undisturbed at room temperature. The crystals were mounted in hydrocarbon oil on a nylon loop or a glass fibre. Low-temperature (110 K) data were collected on an APEX 2-CCD detector equipped SMART 100 Bruker diffractometer with graphite-monochromated Mo Kα radiation (*λ* = 0.71073 Å). Used X-ray data refinement methods have been described previously.^68^ Crystallographic data provided in the form of cif files is available from the CCDC as numbers ; 1483464–; 1483472.^69^ In the case of **5**, the asymmetric unit was found to contain two molecules of **5**, one of which showed a high peak at 1.284 Å from the phosphorus atom. We believe that this peak reflects partial oxidation of the phosphorus atom at this site. It was refined as an oxygen atom with its partial occupancy refining to a value of 22%. In Fig. 5 and in the text, we only discuss the structure of the other independent molecule which shows no such partial oxidation features. Disordered solvent molecules further complicate the structure of this compound.

### Computational details

Density functional theory (DFT) calculations (full geometry optimisation) were carried out on **2**, SbPh_3_(O_2_C_6_Cl_4_), **4**, **5**, **6** and **7** starting from their respective crystal structure geometries with Gaussian09^70^ software (MPW1PW91^71^ functional with 6-31g for H and C; 6-31+G(d′) for O, F and Cl; aug-ccpVTZ for P and Sb;^72^ and Stuttgart relativistic small core ECPs for Sb^73^) using the SMD solvation model for dichloromethane.^74^ Once the optimized structures were in excellent agreement with the observed solid-state structures, frequency calculations were carried out to verify that no imaginary frequencies are present. The coordinates of all these optimized geometries are listed in the ESI.† Wave functions derived from the optimized structures were utilized for QTAIM analysis using the AIMAll^75^ software package. The optimized structures were also subjected to natural bond orbital (NBO)^76^ analysis, and the resulting natural localized molecular orbitals (NLMOs) were plotted using the Jimp 2^77^ software. Plots of the Localized Orbital Locator (LOL), as defined by Becke and Tsirelson,^61^ were visualized and plotted using the Multiwfn software.^61,78^

## References

[cit1] Piers W. E., Chivers T. (1997). Chem. Soc. Rev..

[cit2] Chen E. Y.-X., Marks T. J. (2000). Chem. Rev..

[cit3] Erker G. (2005). Dalton Trans..

[cit4] Piers W. E. (2004). Adv. Organomet. Chem..

[cit5] Piers W. E., Marwitz A. J. V., Mercier L. G. (2011). Inorg. Chem..

[cit6] Stephan D. W. (2015). Acc. Chem. Res..

[cit7] Cardenas A. J. P., Hasegawa Y., Kehr G., Warren T. H., Erker G. (2016). Coord. Chem. Rev..

[cit8] Stephan D. W. (2015). J. Am. Chem. Soc..

[cit9] Stephan D. W., Erker G. (2014). Chem. Sci..

[cit10] Paradies J. (2014). Angew. Chem., Int. Ed..

[cit11] Fontaine F.-G., Courtemanche M.-A., Légaré M.-A. (2014). Chem.–Eur. J..

[cit12] Stephan D. W., Erker G. (2010). Angew. Chem., Int. Ed..

[cit13] Hudnall T. W., Kim Y.-M., Bebbington M. W. P., Bourissou D., Gabbaï F. P. (2008). J. Am. Chem. Soc..

[cit14] Bayne J. M., Stephan D. W. (2016). Chem. Soc. Rev..

[cit15] Robertson A. P. M., Gray P. A., Burford N. (2014). Angew. Chem., Int. Ed..

[cit16] Holthausen M. H., Bayne J. M., Mallov I., Dobrovetsky R., Stephan D. W. (2015). J. Am. Chem. Soc..

[cit17] Holthausen M. H., Mehta M., Stephan D. W. (2014). Angew. Chem., Int. Ed..

[cit18] Caputo C. B., Hounjet L. J., Dobrovetsky R., Stephan D. W. (2013). Science.

[cit19] vom Stein T., Peréz M., Dobrovetsky R., Winkelhaus D., Caputo C. B., Stephan D. W. (2015). Angew. Chem., Int. Ed..

[cit20] Hounjet L. J., Caputo C. B., Stephan D. W. (2012). Angew. Chem., Int. Ed..

[cit21] Krossing I., Raabe I. (2004). Chem.–Eur. J..

[cit22] Ghorab M. F., Winfield J. M. (1990). J. Fluorine Chem..

[cit23] Robertson A. P. M., Chitnis S. S., Jenkins H. A., McDonald R., Ferguson M. J., Burford N. (2015). Chem.–Eur. J..

[cit24] Chitnis S. S., Robertson A. P. M., Burford N., Patrick B. O., McDonald R., Ferguson M. J. (2015). Chem. Sci..

[cit25] Robertson A. P. M., Burford N., McDonald R., Ferguson M. J. (2014). Angew. Chem., Int. Ed..

[cit26] Breunig H. J., Koehne T., Moldovan O., Preda A. M., Silvestru A., Silvestru C., Varga R. A., Piedra-Garza L. F., Kortz U. (2010). J. Organomet. Chem..

[cit27] Jones J. S., Gabbaï F. P. (2016). Acc. Chem. Res..

[cit28] Hirai M., Myahkostupov M., Castellano F. N., Gabbaï F. P. (2016). Organometallics.

[cit29] Jones J. S., Wade C. R., Gabbaï F. P. (2015). Organometallics.

[cit30] Wade C. R., Ke I.-S., Gabbaï F. P. (2012). Angew. Chem., Int. Ed..

[cit31] Fukin G. K., Zakharov L. N., Domrachev G. A., Fedorov A. Y., Zaburdyaeva S. N., Dodonov V. A. (1999). Russ. Chem. Bull..

[cit32] Dodonov V. A., Fedorov A. Y., Fukin G. K., Zaburdyaeva S. N., Zakharov L. N., Ignatenko A. V. (1999). Main Group Chem..

[cit33] Holmes R. R., Day R. O., Chandrasekhar V., Holmes J. M. (1987). Inorg. Chem..

[cit34] Hall M., Sowerby D. B. (1980). J. Am. Chem. Soc..

[cit35] Hirai M., Gabbaï F. P. (2015). Angew. Chem., Int. Ed..

[cit36] Hirai M., Gabbaï F. P. (2014). Chem. Sci..

[cit37] Pan B., Gabbaï F. P. (2014). J. Am. Chem. Soc..

[cit38] Kant R., Singhal K., Shukla S. K., Chandrashekar K., Saxena A. K., Ranjan A., Raj P. (2008). Phosphorus, Sulfur Silicon Relat. Elem..

[cit39] Alvarez S. (2013). Dalton Trans..

[cit40] Zhao X., Lough A. J., Stephan D. W. (2011). Chem.–Eur. J..

[cit41] Yang Z., Ma X., Oswald R. B., Roesky H. W., Zhu H., Schulzke C., Starke K., Baldus M., Schmidt H.-G., Noltemeyer M. (2005). Angew. Chem., Int. Ed..

[cit42] Chen J., Chen E. Y. X. (2016). Dalton Trans..

[cit43] Müller L. O., Himmel D., Stauffer J., Steinfeld G., Slattery J., Santiso-Quiñones G., Brecht V., Krossing I. (2008). Angew. Chem., Int. Ed..

[cit44] Sarazin Y., Hughes D. L., Kaltsoyannis N., Wright J. A., Bochmann M. (2007). J. Am. Chem. Soc..

[cit45] Díez Á., Fernández J., Lalinde E., Moreno M. T., Sánchez S. (2010). Inorg. Chem..

[cit46] Takemura H., Nakashima S., Kon N., Yasutake M., Shinmyozu T., Inazu T. (2001). J. Am. Chem. Soc..

[cit47] Arp H., Baumgartner J., Marschner C., Müller T. (2011). J. Am. Chem. Soc..

[cit48] Romanato P., Duttwyler S., Linden A., Baldridge K. K., Siegel J. S. (2011). J. Am. Chem. Soc..

[cit49] García-Monforte M. A., Alonso P. J., Ara I., Menjón B., Romero P. (2012). Angew. Chem., Int. Ed..

[cit50] Gutmann V. (1976). Coord. Chem. Rev..

[cit51] Beckett M. A., Brassington D. S., Coles S. J., Hursthouse M. B. (2000). Inorg. Chem. Commun..

[cit52] Pyykkö P., Atsumi M. (2009). Chem.–Eur. J..

[cit53] Arduengo A. J., Stewart C. A., Davidson F., Dixon D. A., Becker J. Y., Culley S. A., Mizen M. B. (1987). J. Am. Chem. Soc..

[cit54] Chitnis S. S., Vos K. A., Burford N., McDonald R., Ferguson M. J. (2016). Chem. Commun..

[cit55] Courtemanche M.-A., Légaré M.-A., Maron L., Fontaine F.-G. (2013). J. Am. Chem. Soc..

[cit56] Courtemanche M.-A., Légaré M.-A., Maron L., Fontaine F.-G. (2014). J. Am. Chem. Soc..

[cit57] Declercq R., Bouhadir G., Bourissou D., Légaré M.-A., Courtemanche M.-A., Nahi K. S., Bouchard N., Fontaine F.-G., Maron L. (2015). ACS Catal..

[cit58] Levason W., McAuliffe C. A. (1974). Inorg. Chim. Acta.

[cit59] Lin T.-P., Gualco P., Ladeira S., Amgoune A., Bourissou D., Gabbaï F. P. (2010). C. R. Chim..

[cit60] Chalmers B. A., Bühl M., Athukorala Arachchige K. S., Slawin A. M. Z., Kilian P. (2015). Chem.–Eur. J..

[cit61] Lu T., Chen F. (2012). J. Comput. Chem..

[cit62] Bader R. F. W., Stephens M. E. (1975). J. Am. Chem. Soc..

[cit63] Macchi P., Sironi A. (2003). Coord. Chem. Rev..

[cit64] National Cancer Institute, Formaldehyde and Cancer Risk, http://www.goo.gl/Wr1Dqk, accessed May 1, 2016.

[cit65] Mo Z., Kolychev E. L., Rit A., Campos J., Niu H., Aldridge S. (2015). J. Am. Chem. Soc..

[cit66] De Crisci A. G., Lough A. J., Multani K., Fekl U. (2008). Organometallics.

[cit67] Budzelaar P. H. M., gNMR, version 5.1, http://www.home.cc.umanitoba.ca/∼budzelaa/gNMR/gNMR.html.

[cit68] Tofan D., Cossairt B. M., Cummins C. C. (2011). Inorg. Chem..

[cit69] Dawson W. R., Windsor M. W. (1968). J. Phys. Chem..

[cit70] Frisch M. J., Trucks G. W., Schlegel H. B., Scuseria G. E., Robb M. A., Cheeseman J. R., Scalmani G., Barone V., Mennucci B., Petersson G. A., Nakatsuji H., Caricato M., Li X., Hratchian H. P., Izmaylov A. F., Bloino J., Zheng G., Sonnenberg J. L., Hada M., Ehara M., Toyota K., Fukuda R., Hasegawa J., Ishida M., Nakajima T., Honda Y., Kitao O., Nakai H., Vreven T., Montgomery J., Peralta J. E., Ogliaro F., Bearpark M., Heyd J. J., Brothers E., Kudin K. N., Staroverov V. N., Kobayashi R., Normand J., Raghavachari K., Rendell A., Burant J. C., Iyengar S. S., Tomasi J., Cossi M., Rega N., Millam J. M., Klene M., Knox J. E., Cross J. B., Bakken V., Adamo C., Jaramillo J., Gomperts R., Stratmann R. E., Yazyev O., Austin A. J., Cammi R., Pomelli C., Ochterski J. W., Martin R. L., Morokuma K., Zakrzewski V. G., Voth G. A., Salvador P., Dannenberg J. J., Dapprich S., Daniels A. D., Farkas Ö., Foresman J. B., Ortiz J. V., Cioslowski J. and Fox D. J., Gaussian 09, Revision B.01, Gaussian, Inc., Wallingford, CT, 2009.

[cit71] Adamo C., Barone V. (1998). J. Chem. Phys..

[cit72] Peterson K. A. (2003). J. Chem. Phys..

[cit73] Metz B., Stoll H., Dolg M. (2000). J. Chem. Phys..

[cit74] Marenich A. V., Cramer C. J., Truhlar D. G. (2009). J. Phys. Chem. B.

[cit75] T. A. Keith, AIMAll, Version 16.05.18, TK Gristmill Software, Overland Park KS, USA, 2016 (aim.tkgristmill.com).

[cit76] Glendening E. D., Badenhoop J. K., Reed A. E., Carpenter J. E., Bohmann J. A., Morales C. M. and Weinhold F., NBO 5.9, Theoretical Chemistry Institute, University of Wisconsin, Madison, WI, 2011.

[cit77] Manson J., Webster C. E., Pérez L. M. and Hall M. B., Jimp 2, Version 091, 2006http://www.chem.tamu.edu/jimp2/index.html.

[cit78] Lu T., Chen F. (2012). J. Mol. Graphics Modell..

