# Supplementary material for: Fluorinated antimony(v) derivatives: strong Lewis acidic properties and application to the complexation of formaldehyde in aqueous solutions
Source: Chem Sci. 2016 Jul 11;7(11):6768–78. doi: 10.1039/c6sc02558g (PMC5363782; doi:10.1039/c6sc02558g)
Supplement: Supplementary file 1 [file SC-007-C6SC02558G-s001.pdf]

Electronic Supplementary Material (ESI) for Chemical Science.
This journal is © The Royal Society of Chemistry 2016

Fluorinated Antimony(V) Derivatives: Strong Lewis Acidic Properties and Application to the Complexation of Formaldehyde in Aqueous Solutions

Supporting Information

Daniel Tofan, Francois P. Gabbaï*

Department of Chemistry, Texas A&M University, College Station, Texas 77843-3255, United States

E-mail: francois@tamu.edu

*To whom the correspondence should be addressed

Table of Contents

S.1 Optimized protocol for the synthesis of $Sb(C_6F_5)_3$ (**1**)

S.2 Stiborane $Sb(C_6F_5)_3(O_2C_6Cl_4)$ (**2**)

S.3 Treatment of **2** and $SbPh_3(O_2C_6Cl_4)$ with $OPEt_3$

S.4 Complex $Sb(C_6F_5)_3(O_2C_6Cl_4)(OCH_2)(P^tBu_3)$ (**3**)

S.5 Stiborane (o-C_6H_4)(PPh_2)($SbPh_2(O_2C_6Cl_4)$) (**5**)

S.6 Stibine (o-C_6H_4)(PPh_2)($Sb(C_6F_5)_2$) (**6**)

S.7 Stiborane (o-C_6H_4)(PPh_2)($Sb(C_6F_5)_2(O_2C_6Cl_4)$) (**7**)

S.8 Complex (o-C_6H_4)(PPh_2)($SbPh_2(O_2C_6Cl_4)$)(CH_2O) (**8**)

S.9 Complex (o-C_6H_4)(PPh_2)($Sb(C_6F_5)_2(O_2C_6Cl_4)$)(CH_2O) (**9**)

S.10 Detection of formaldehyde from aqueous solutions with stiborane **7**

S.11 Computational details

S.1 Optimized protocol for the synthesis of $Sb(C_6F_5)_3$ (1)

In a modified protocol of the reported synthesis of **1**, a Schlenk flask containing diethyl ether (120 mL), magnesium granules (2.8 g, 0.115 mol, 3.2 equiv), and a crystal of elemental iodine was placed in a bath at 0 °C. C_6F_5Br (29.9 g, 0.109 mol, 3.0 equiv) was added drop-wise *via* a syringe. The resulting mixture was stirred for 90 min, and then cooled to −10 °C. $SbCl_3$ (8.4 g, 0.037 mol, 1.02 equiv) in diethyl ether (35 mL) was added drop-wise over 30 min, and the resulting stirring mixture was allowed to warm up over the next 16 h. After adding aqueous HCl (40 mL, 0.5 M, 0.02 mol), the mixture was extracted with diethyl ether. $MgSO_4$ and charcoal were added to the organic extract, and filtration through Celite yielded a brown filtrate. Volatiles were removed under reduced pressure and the residue was redissolved in hexanes and passed through a short (2 cm) column of silica and washed with hexanes (200 mL). Volatiles were removed again under reduced pressure to yield a colorless oil which solidified into a white crystalline solid upon sitting, and consisted of analytically pure 1 (17.7 g, 28.4 mmol, 78% yield). ^{19}F NMR ($CDCl_3$, 20 °C, 375.9 MHz) δ: −118.8 (d, $^3J_{FF}$ = 19 Hz), −145.0 (t, $^3J_{FF}$ = 20 Hz), −155.5 (t, $^3J_{FF}$ = 19 Hz) ppm. $^{13}C\{^1H\}$ NMR ($CDCl_3$, 20 °C, 100.5 MHz) δ: 148.4 (br d, $^1J_{CF}$ = 240 Hz, *o*), 143.1 (br d, $^1J_{CF}$ = 255Hz, *p*), 137.5 (ddd,$^1J_{CF}$ =255Hz, $^3J_{CF}$ = 23 Hz, $^3J_{CF}$ = 17 Hz, *m*), 104.1(br t, $^2J_{CF}$ = 33 Hz, *i*) ppm. Elemental analysis found (calcd. for $C_{18}F_{15}Sb$) [%]: C 34.70 (34.71), H 0.00 (0.00).

S.2 Stiborane $Sb(C_6F_5)_3(O_2C_6Cl_4)$ (2)

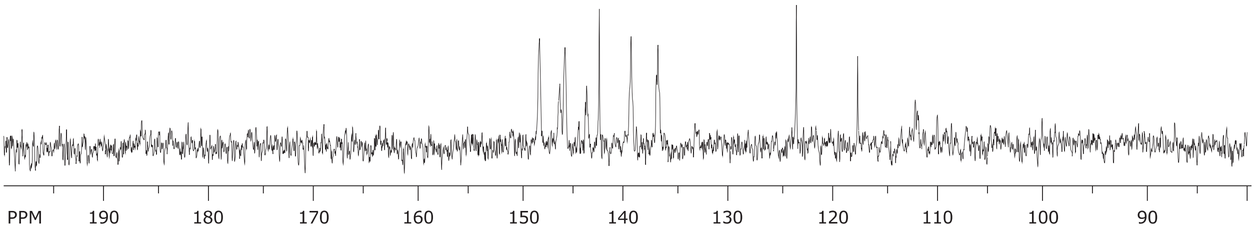

Figure S.1. $^{13}C\{^1H\}$ NMR spectrum of **2** in $CDCl_3$

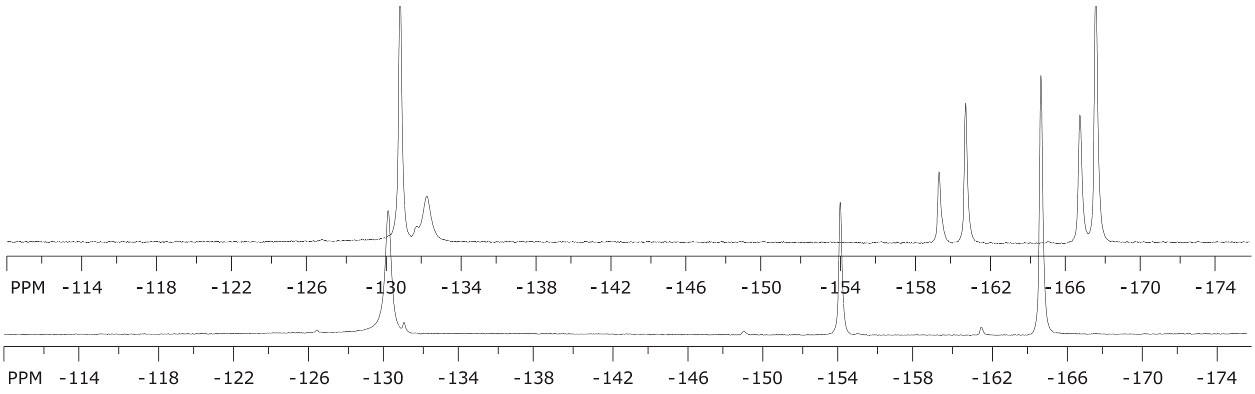

Figure S.2. ^{19}F NMR spectra of **2** after 3 days in solution of a 1:1 THF/H_2O mixture (top) *vs* in a solution of unpurified THF (bottom).

S.3 Treatment of 2 and $SbPh_3(O_2C_6Cl_4)$ with Et_3PO

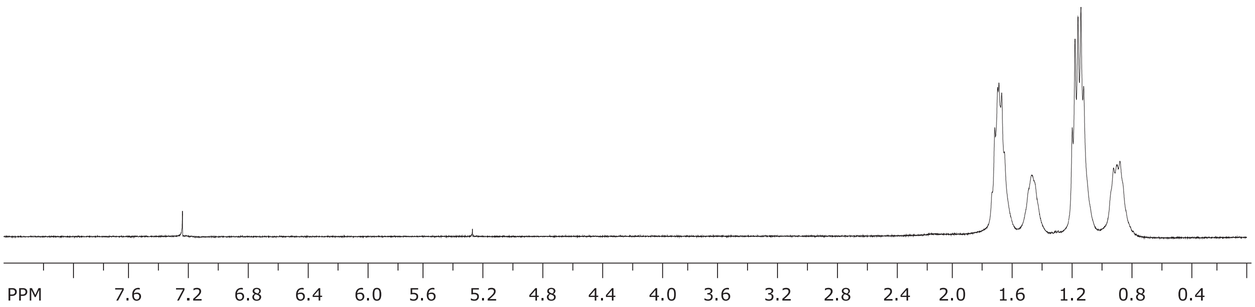

Figure S.3. ^{1}H NMR spectrum of a 1:3 mixture of **2**/Et_3PO showing sharp multiplets for free Et_3PO and broad peaks for the $Et_3PO{\cdot}$**2** adduct

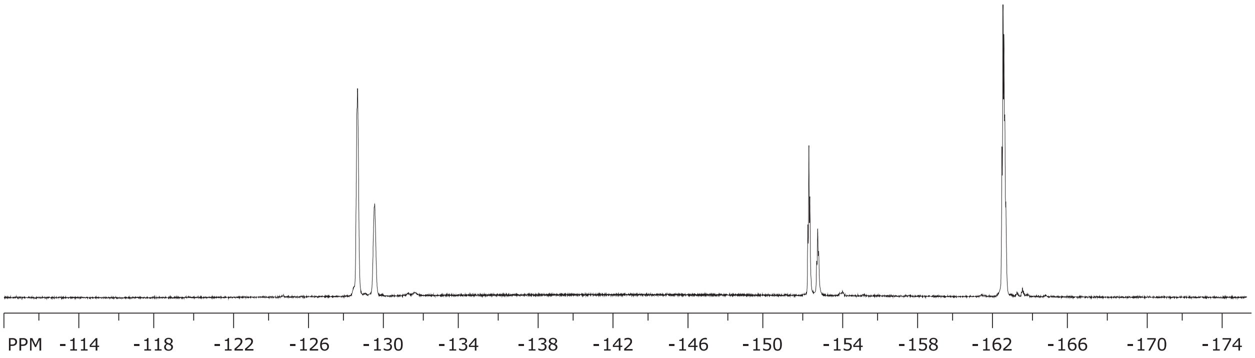

Figure S.4. ^{19}F NMR spectrum of the $Et_3PO{\cdot}$**2** adduct

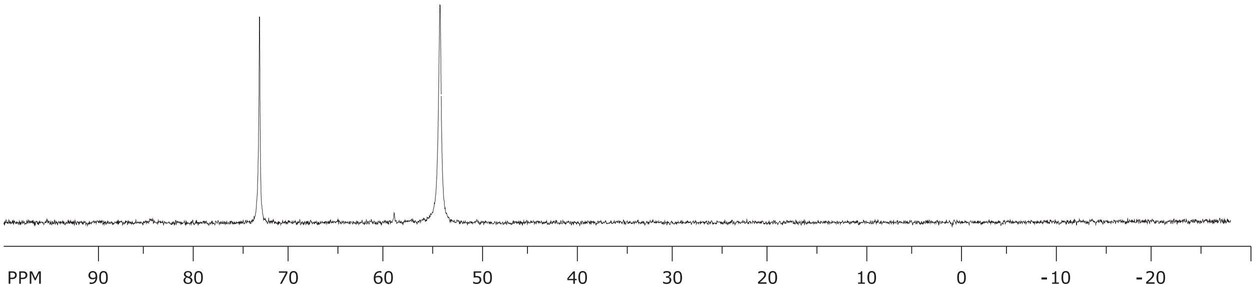

Figure S.5. $^{31}P\{^{1}H\}$ NMR spectrum of a 1:3 mixture of **2**/Et_3PO showing the resonances for $Et_3PO{\cdot}$**2** adduct and free Et_3PO

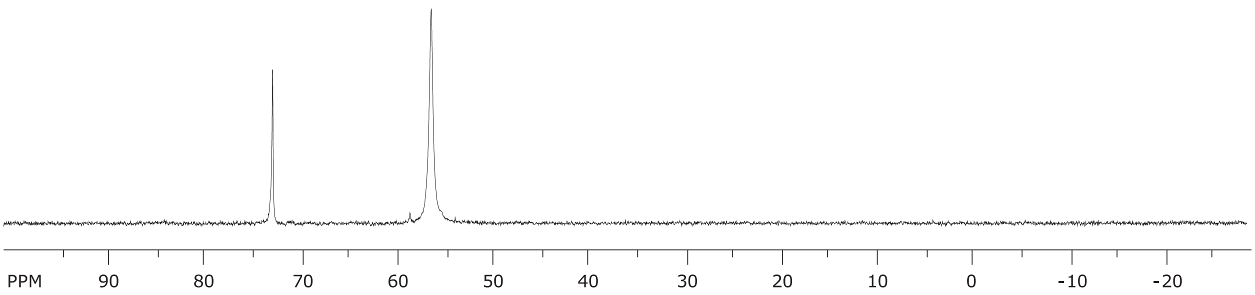

Figure S.6. ^{31}P NMR spectrum of a 1:1:5 mixture of 2 /$SbPh_3(O_2C_6Cl_4)$/Et_3PO the resonance of the Et_3PO·**2** adduct and the averaged-out peak of the $(Et_3PO)SbPh_3(O_2C_6Cl_4)$ adduct and free Et_3PO

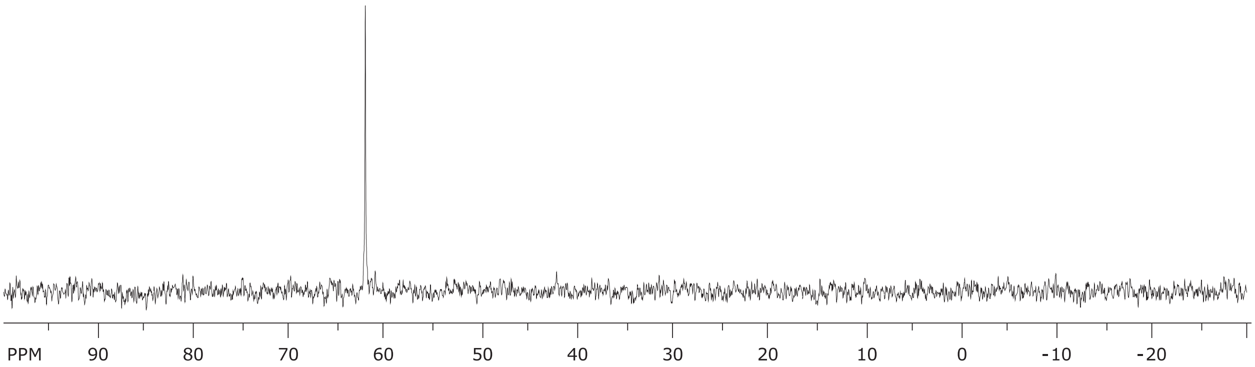

Figure S.7. ^{19}F NMR spectrum of a 10:1 mixture of $SbPh_3(O_2C_6Cl_4)$/Et_3PO in $CDCl_3$

S.4 Complex $Sb(C_6F_5)_3(O_2C_6Cl_4)(OCH_2)(P^tBu_3)$ (3)

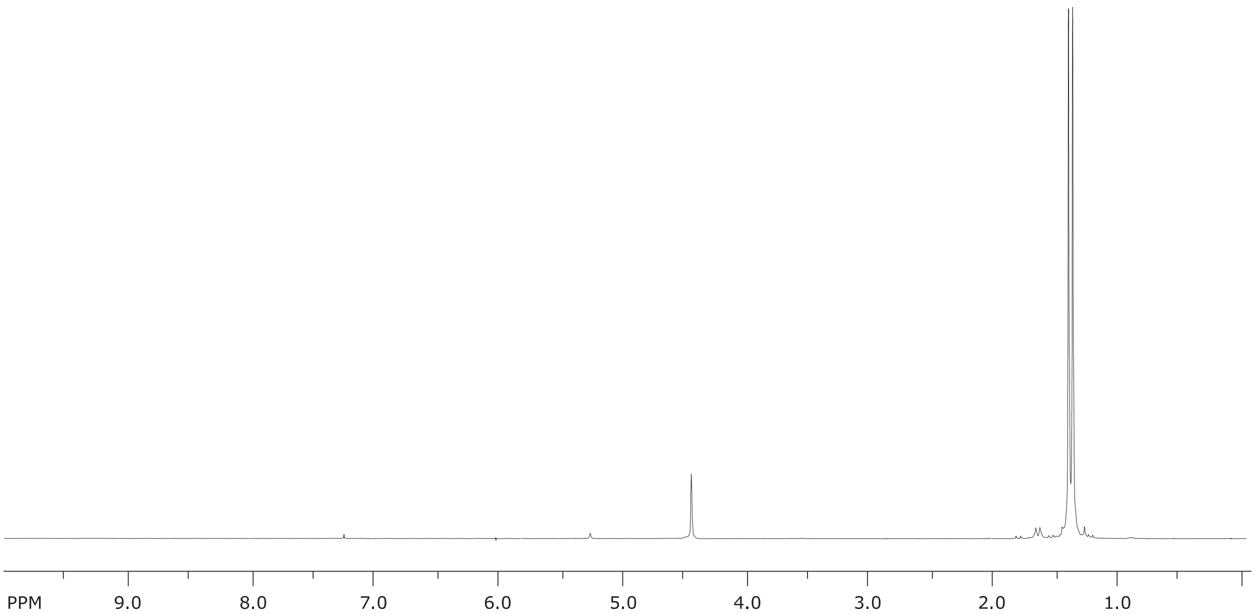

Figure S.8. ^1H NMR spectrum of **3** in $CDCl_3$

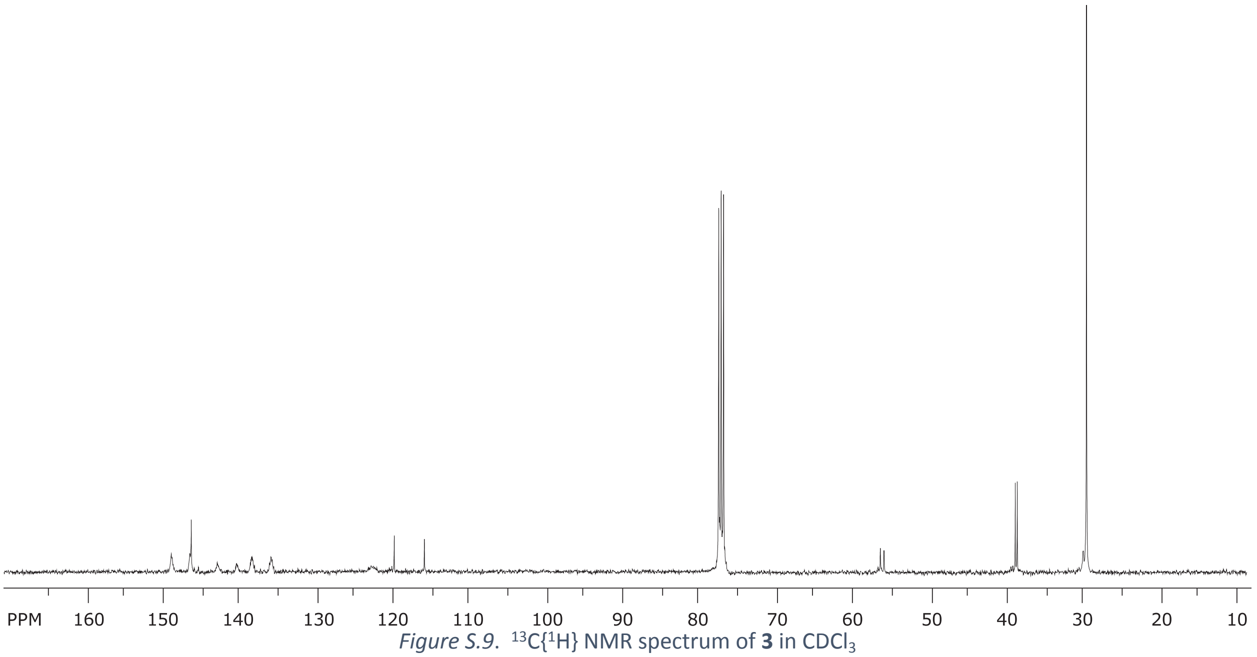

Figure S.9. $^{13}C\{^1H\}$ NMR spectrum of **3** in $CDCl_3$

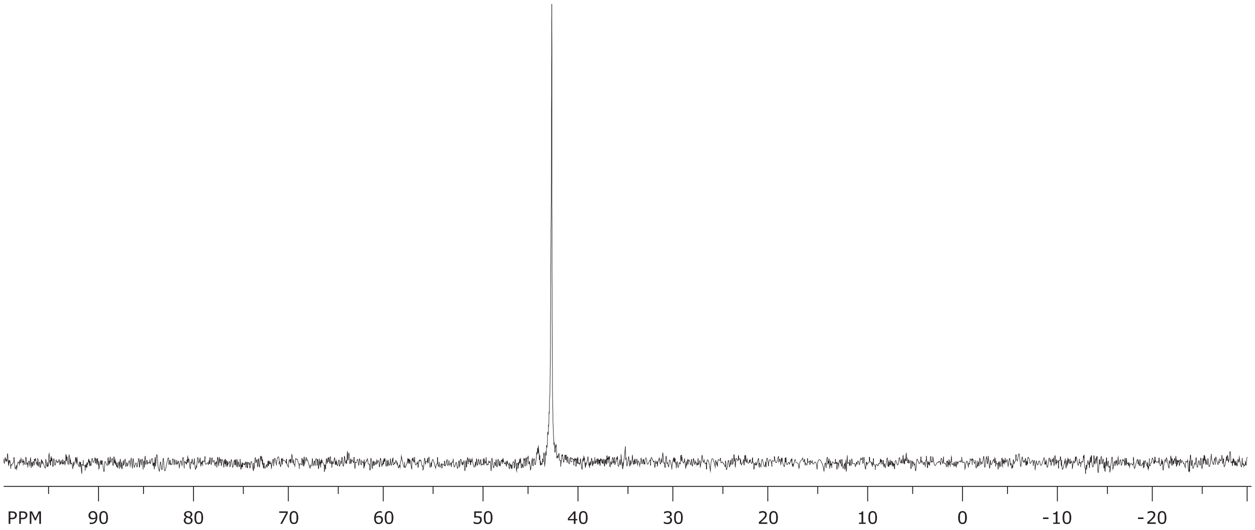

Figure S.10. $^{31}P\{^1H\}$ NMR spectrum of **3** in $CDCl_3$

S.5 Stiborane (o-C_6H_4)(PPh_2)($SbPh_2(O_2C_6Cl_4)$) (5)

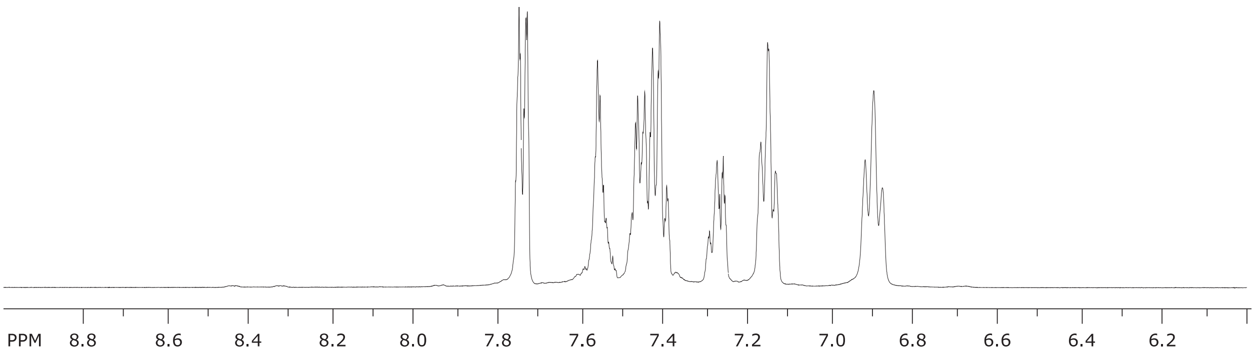

Figure S.11. ^1H NMR spectrum of **5** in $CDCl_3$

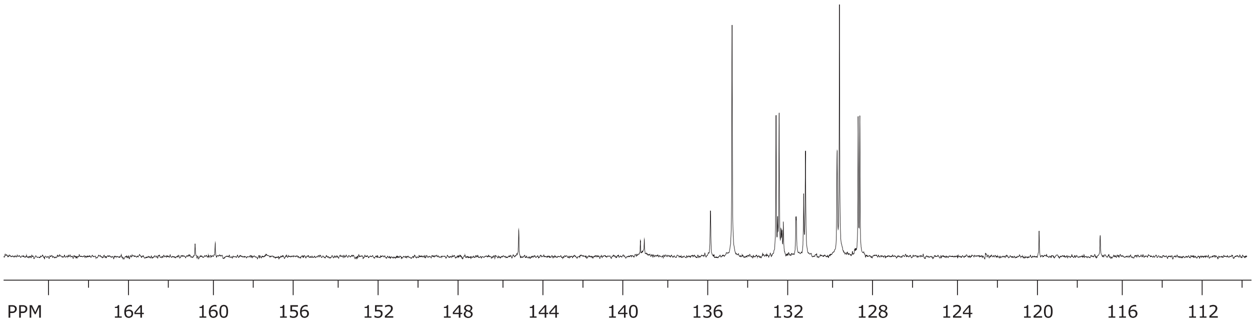

Figure S.12. $^{13}C\{^1H\}$ NMR spectrum of **5** in $CDCl_3$

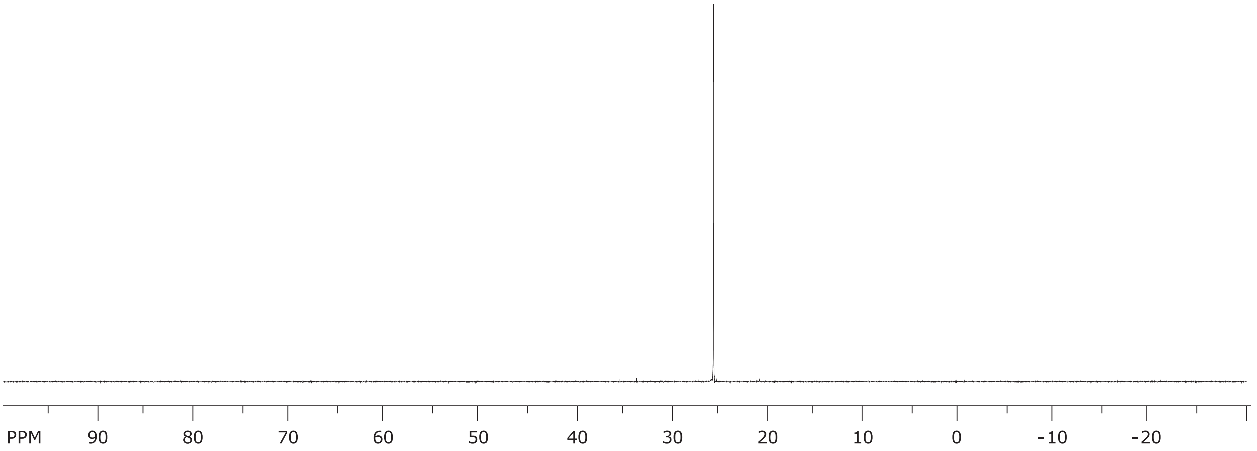

Figure S.13. $^{31}P\{^1H\}$ NMR spectrum of **5** in $CDCl_3$

S.6 Stibine (o-C_6H_4)(PPh_2)($Sb(C_6F_5)_2$) (6)

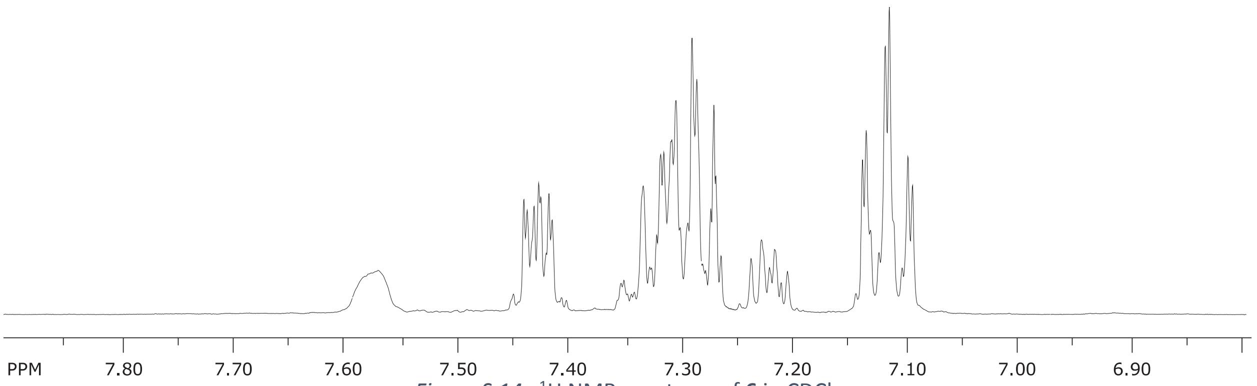

Figure S.14. ^{1}H NMR spectrum of **6** in $CDCl_3$

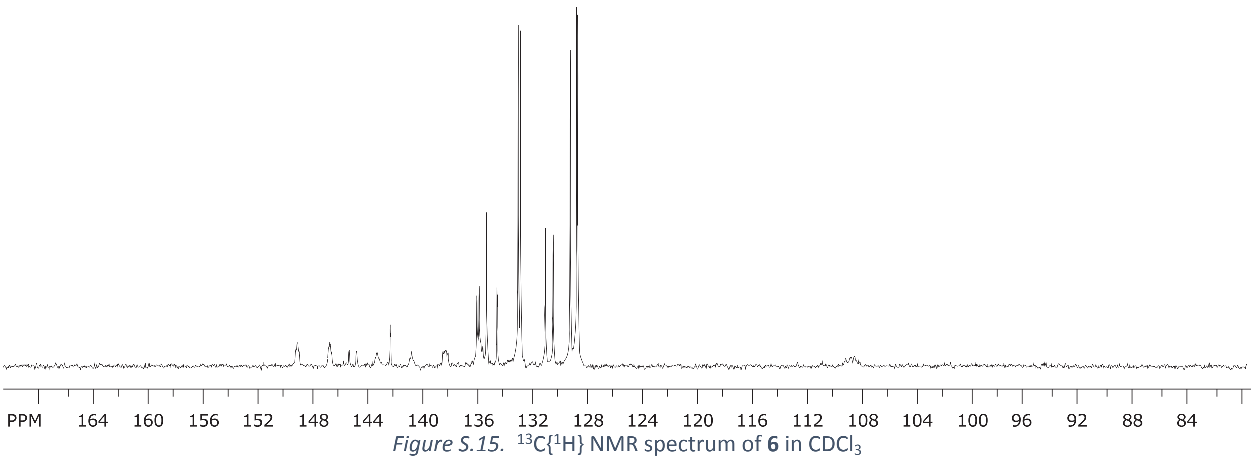

Figure S.15. $^{13}C\{^{1}H\}$ NMR spectrum of **6** in $CDCl_3$

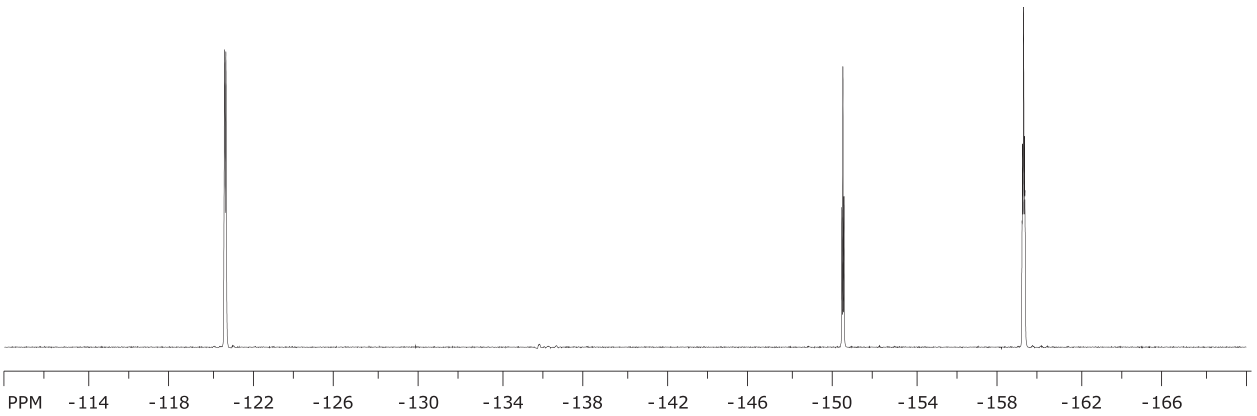

Figure S.16. ^{19}F NMR spectrum of **6** in $CDCl_3$

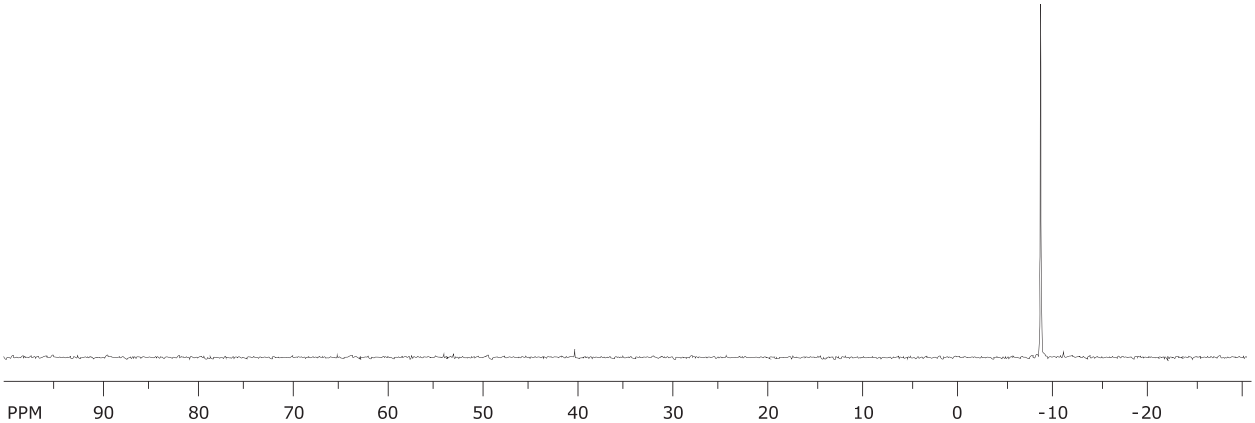

Figure S.17. $^{31}P\{^1H\}$ NMR spectrum of **6** in $CDCl_3$

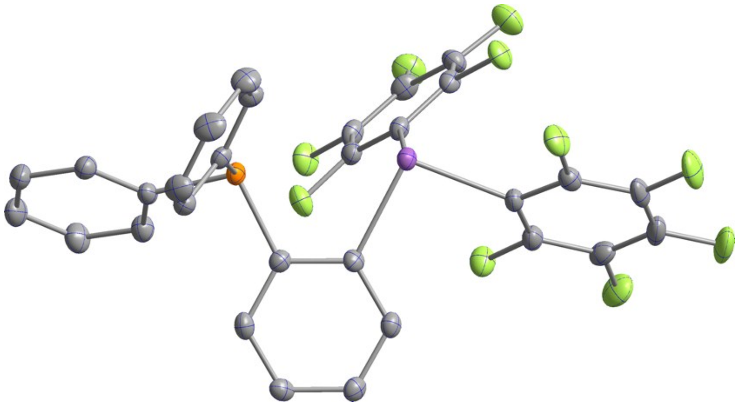

Figure S.18: Solid-state structure of **6** (CSD #1483468) with thermal ellipsoids drawn at the 50% probability level. Selected distances [Å] and angles [°]: Sb–P 3.2332(9), Sb–C(11) 2.159(3), Sb–C(41) 2.174(3), Sb–C(51) 2.199(3), P–C(21) 1.829(3), P–C(31) 1.834(3), P–C(12) 1.838(3), C(11)–C(12)–P 116.4(2), C(12)–C(11)–Sb 117.5(2).

S.7 Stiborane (o-C_6H_4)(PPh_2)($Sb(C_6F_5)_2(O_2C_6Cl_4)$) (7)

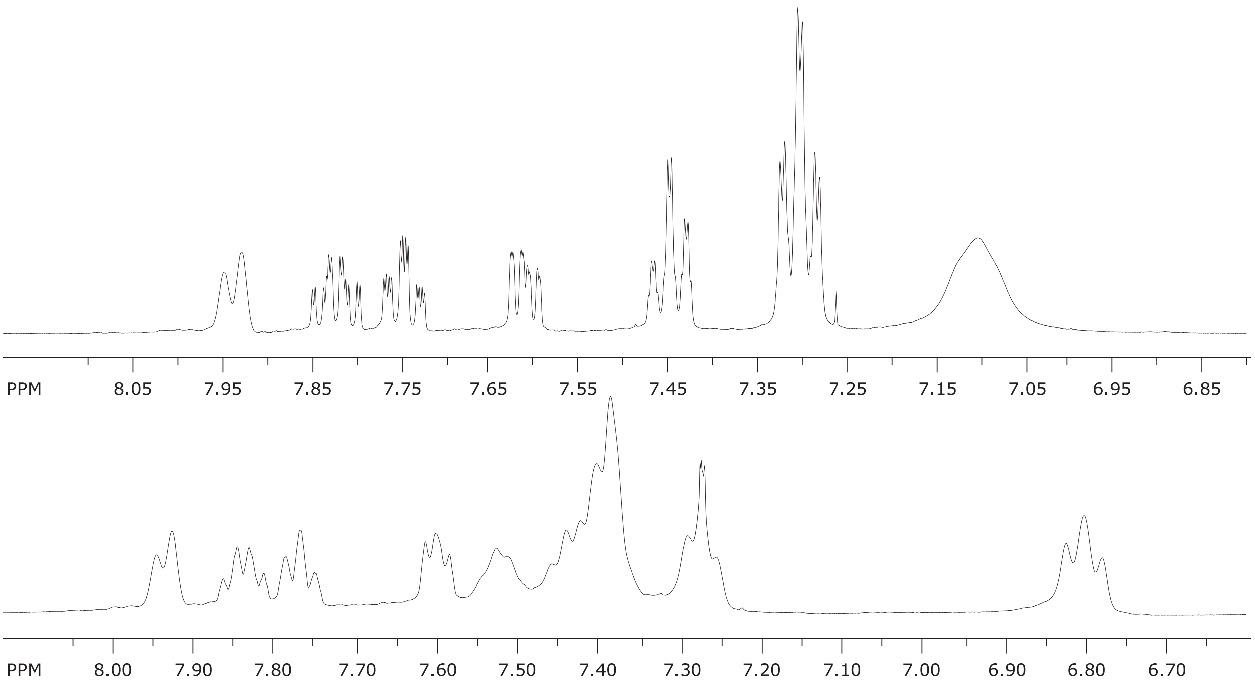

Figure S.19. ^{1}H NMR spectra of **7** in $CDCl_3$ at +20 °C (top) and at −50 °C (bottom)

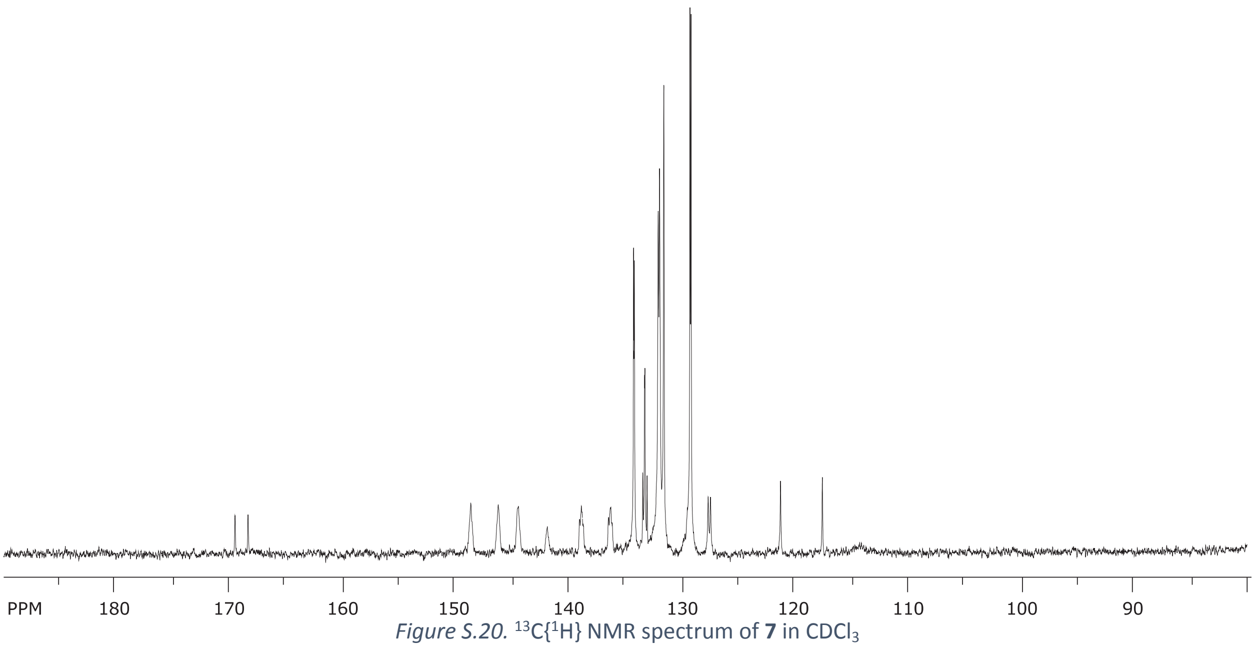

Figure S.20. $^{13}C\{^1H\}$ NMR spectrum of **7** in $CDCl_3$

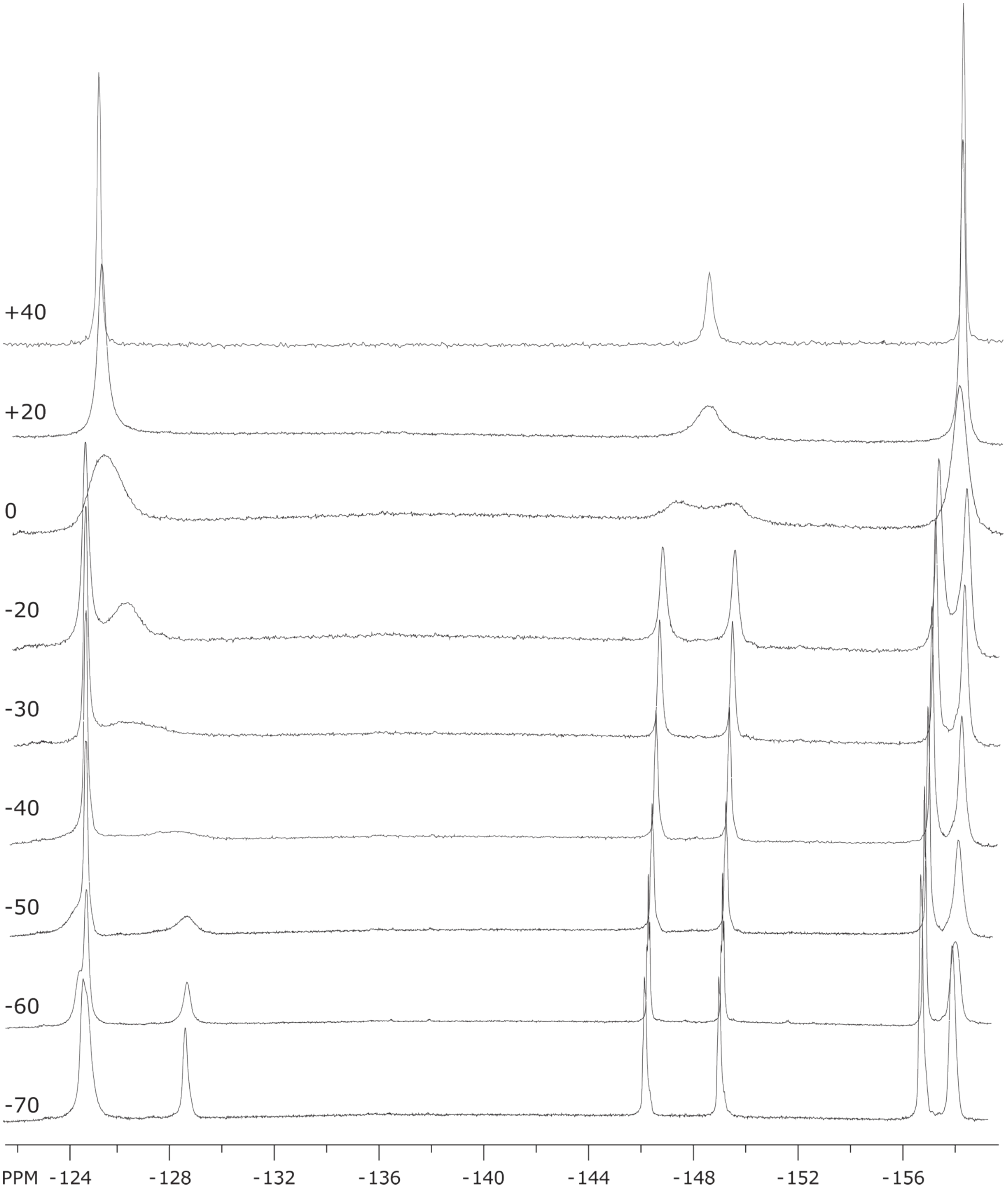

Figure S.21. ^{19}F NMR spectra of **7** in $CDCl_3$ obtained between +40 °C and –70 °C. Steric hindrance from the nearby phenylene hydrogen atom leads to two distinct chemical environments for one the C_6F_5 groups (likely the group trans to the phosphorus atom). The high concentration of the sample used allowed for data points at temperatures below the freezing point of the pure solvent.

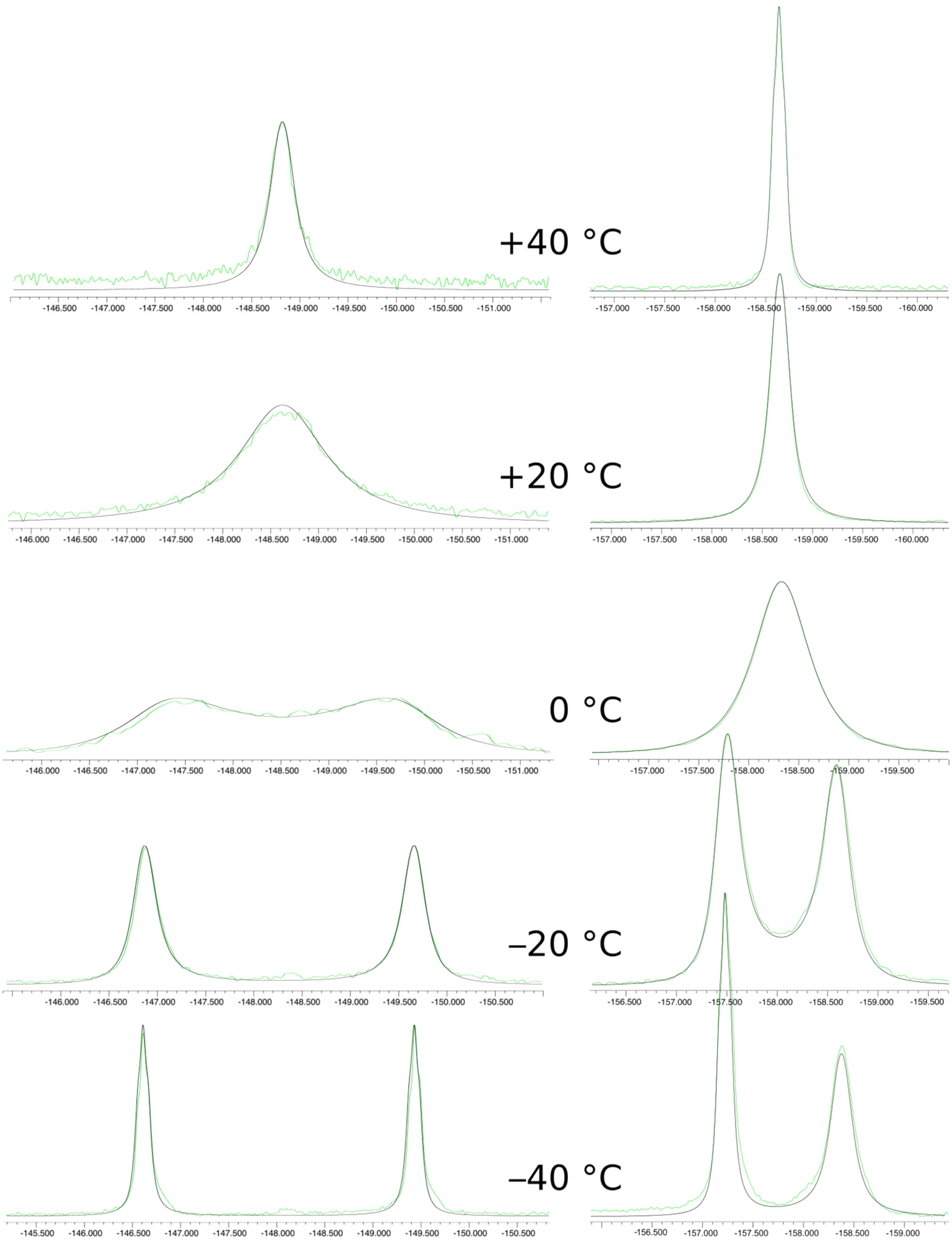

Figure S.22. Experimental (green) and *gNMR* fits (black) of the ^{19}F NMR signals of the *para* (left) and *meta* (right) fluorine atoms of **5** in $CDCl_3$,. Additional broadening in the upfield *meta* signal is due to additional splitting in one of the C_6F_5 groups.

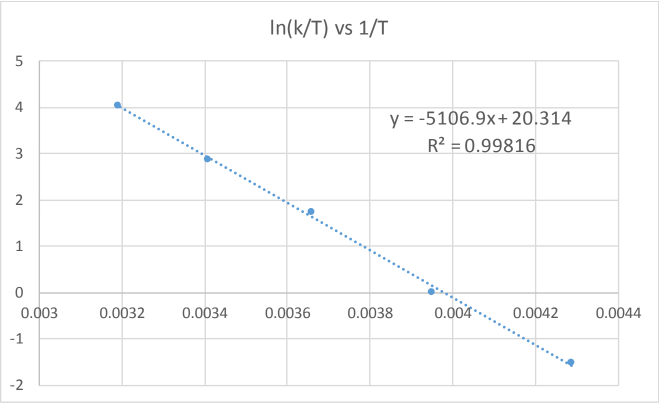

T (°C)	k_{fit} (s^{-1})
40	17500
20	5000
0	1500
−20	250
−40	50

$\Delta H^{\ddagger} = 10.15$ kcal mol^{-1}

$\Delta S^{\ddagger} = -6.85$ cal mol^{-1} K^{-1}

Figure S.23. Eyring plot of ln(k_{fit})/T *vs* 1/T

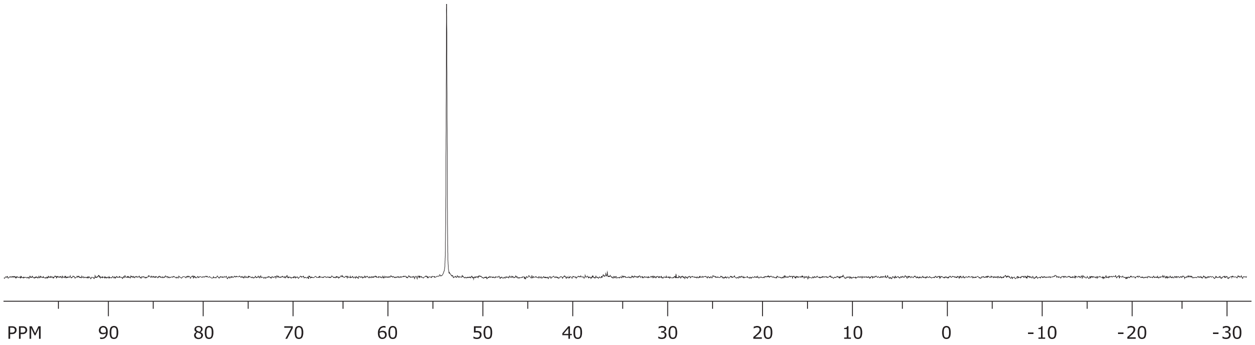

Figure S.24. $^{31}P\{^{1}H\}$ NMR spectrum of **7** in $CDCl_3$ at +20 °C

S.8 Complex (o-C_6H_4)(PPh_2)($SbPh_2(O_2C_6Cl_4)$)(CH_2O) (8)

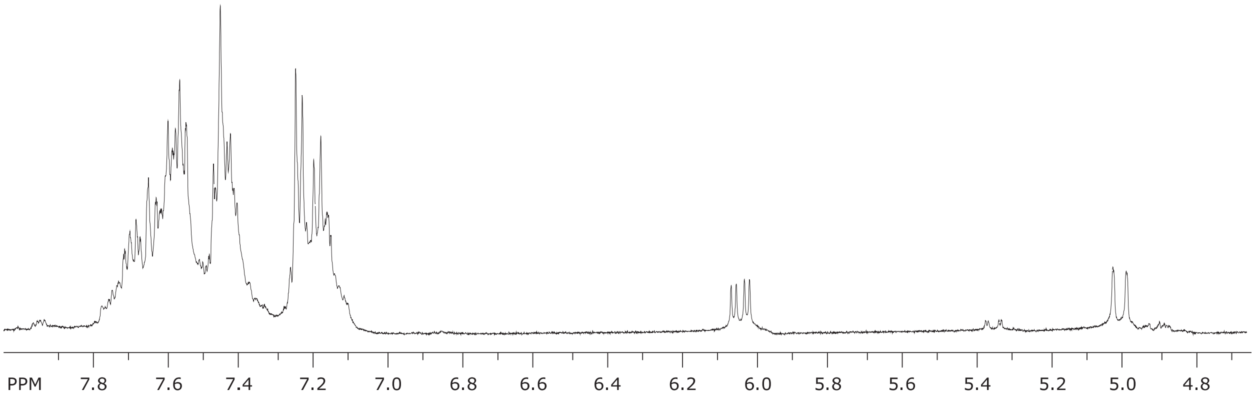

Figure S.25. ^{1}H NMR spectrum of **8** in $CDCl_3$

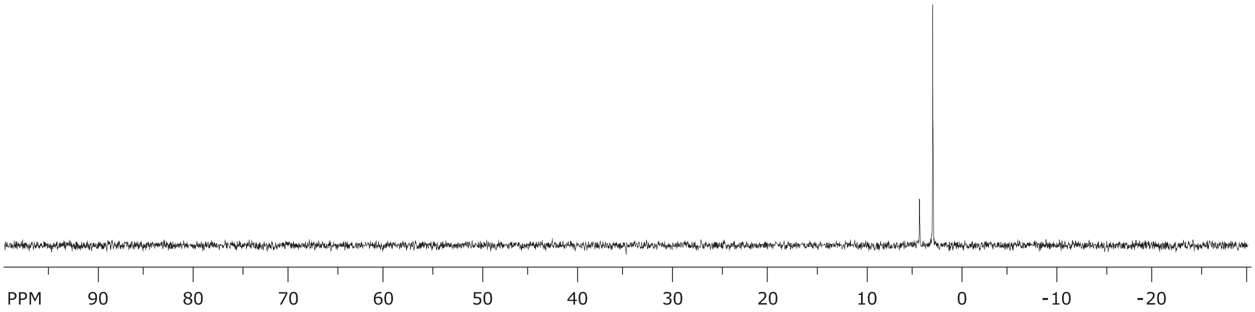

Figure S.26. $^{31}P\{^{1}H\}$ NMR spectrum of **8** in $CDCl_3$

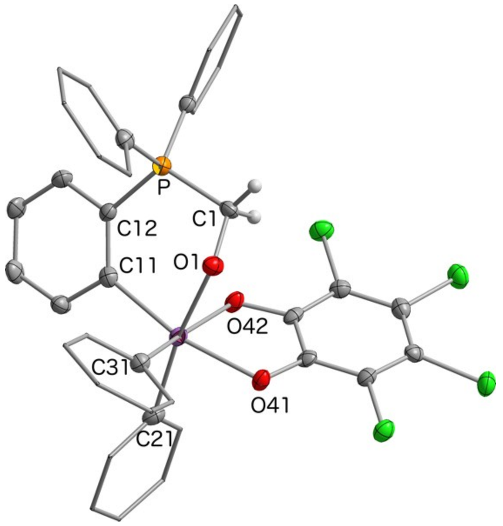

Figure S.27. Solid-state structure of the major isomer of **8** (CSD #1483470) with thermal ellipsoids drawn at the 50% probability level. Phenyl groups are drawn in wireframe, while hydrogen atoms (barring methylene H) and solvent molecules omitted for clarity. Selected distances [Å] and angles [°]: Sb–O(1) 2.044(3), Sb–O(41) 2.080(3), Sb–O(42) 2.082(3), Sb–C(11) 2.168(5), P–C(12) 1.793(5), P–C(1) 1.800(5), O(1)–C(1) 1.393(5), O(42)–Sb–C(31) 164.58(15), O(1)–Sb–C(21) 172.50(15), O(41)–Sb–C(11) 163.76(14), C(1)–O(1)–Sb 122.2(3), O(1)–C(1)–P 110.8(3).

S.9 Complex (o-C_6H_4)(PPh_2)($Sb(C_6F_5)_2(O_2C_6Cl_4)$)(CH_2O) (9)

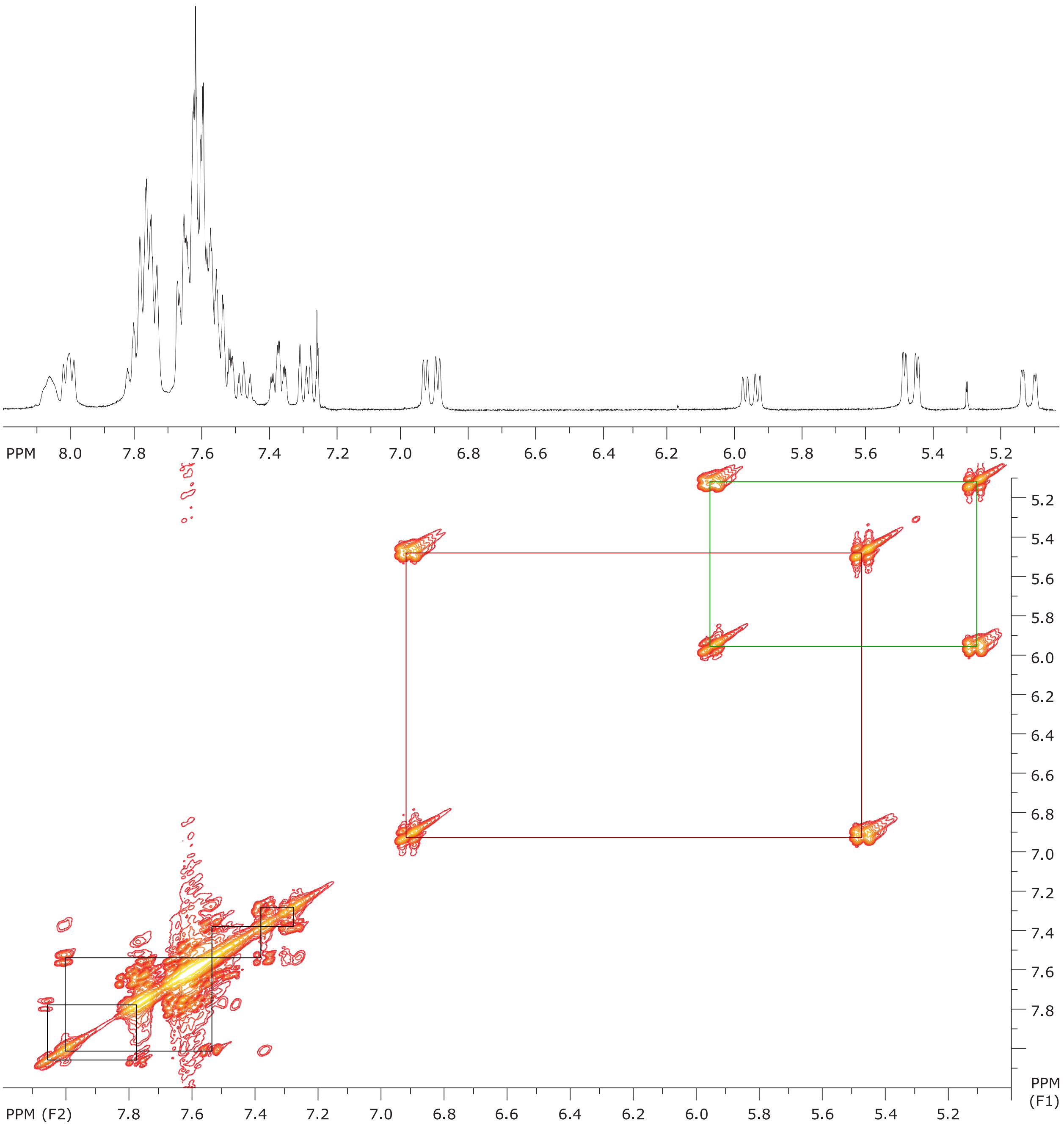

Figure S.28. ^{1}H and ^{1}H-^{1}H COSY NMR spectra of **9** in $CDCl_3$ showing the two isomers in a 35:65 ratio.

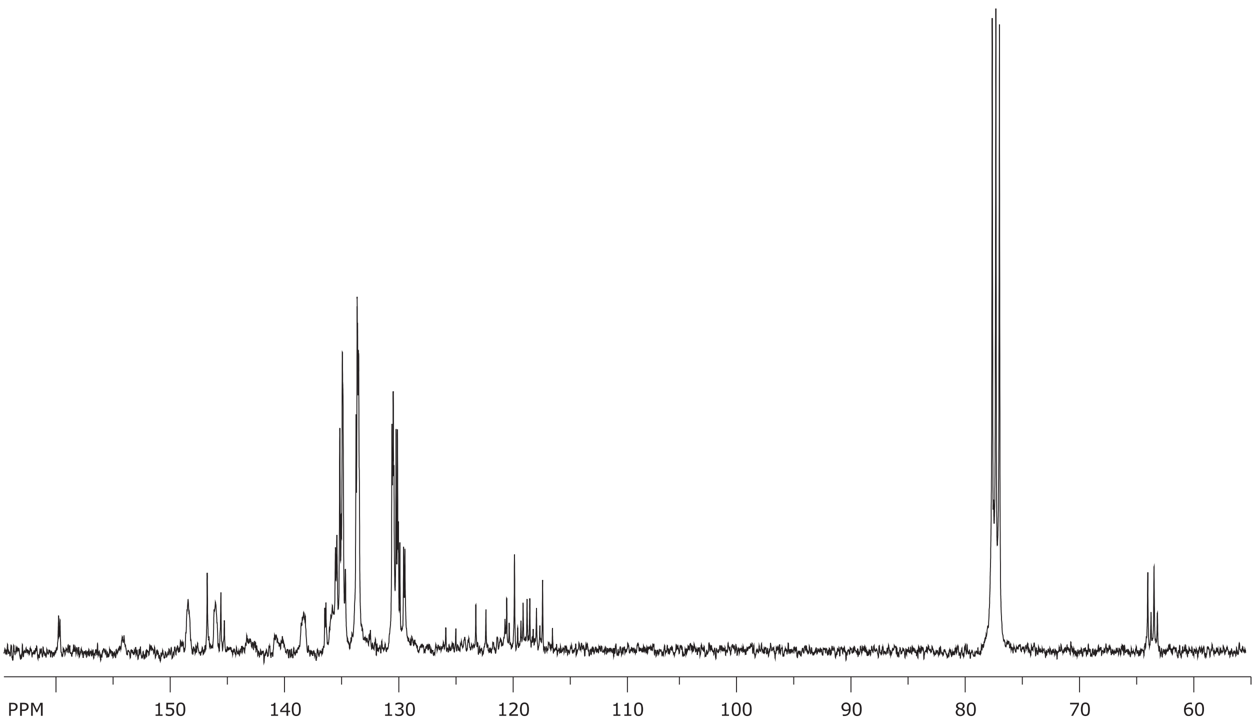

Figure S.29. $^{13}C\{^{1}H\}$ NMR spectrum of **9** in $CDCl_3$ at +40 °C showing two doublets at 63 ppm ($^{1}J_{CP}$ = 56 Hz).

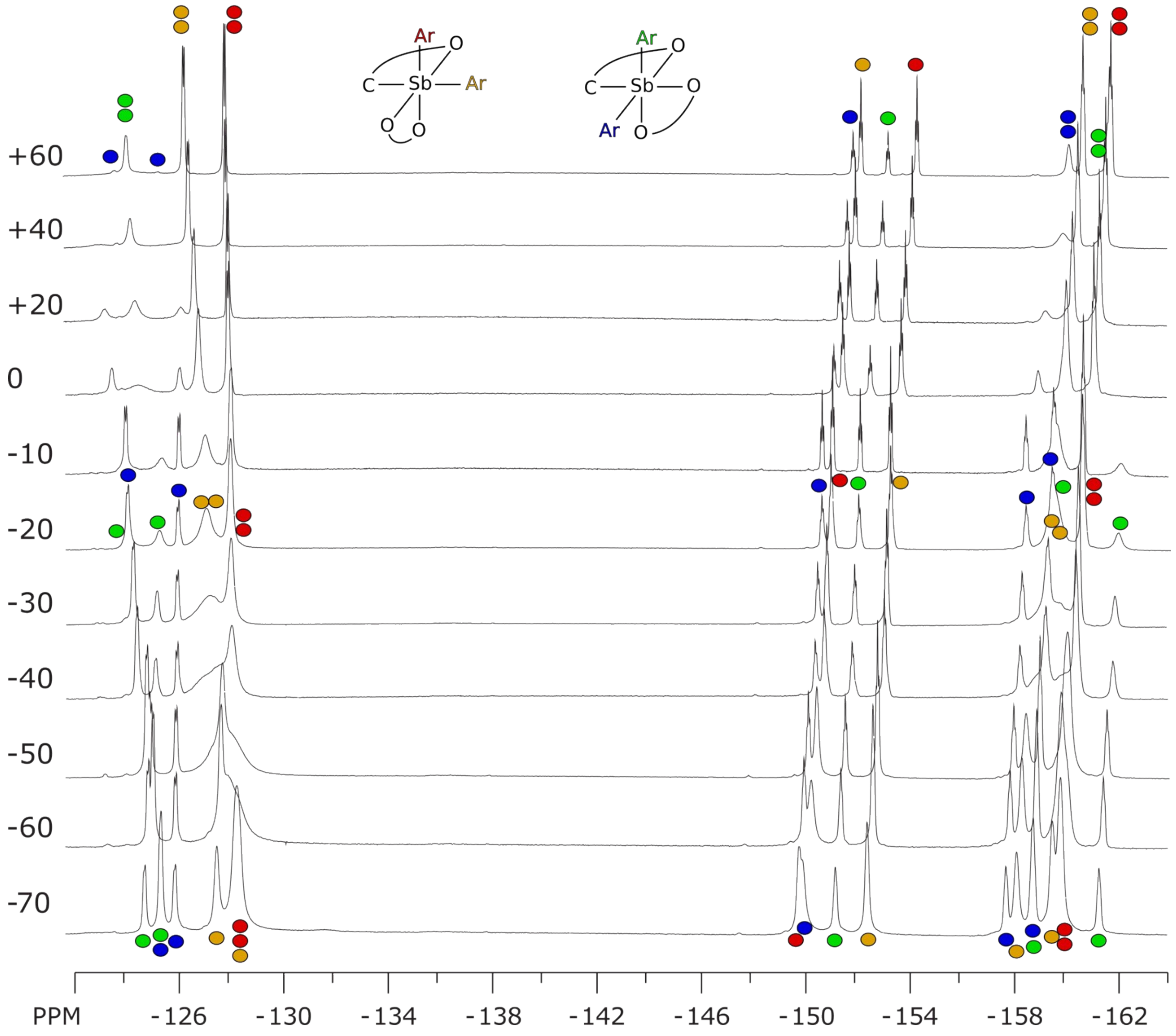

Figure S.30. ^{19}F NMR spectra of **9** in $CDCl_3$ obtained between +60 °C and –70 °C. Due to steric hindrance from other ligands (likely phenylene hydrogen and formaldehyde oxygen), three of the four C_6F_5 groups split into two separate environments (blue below +40 °C., green below 0 °C. orange below –20 °C). The high concentration of the sample used allowed for data points at temperatures below the freezing point of the pure chloroform.

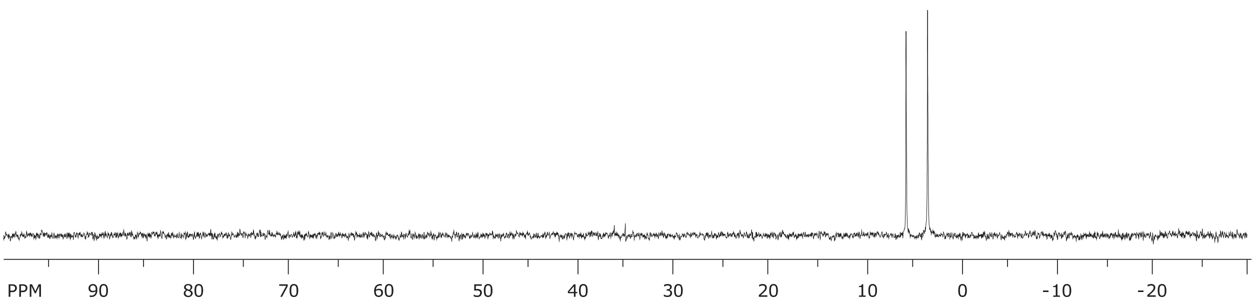

Figure S.31. $^{31}P\{^1H\}$ NMR spectrum of **9** in $CDCl_3$

S.10 Detection of formaldehyde from aqueous solutions with stiborane 7

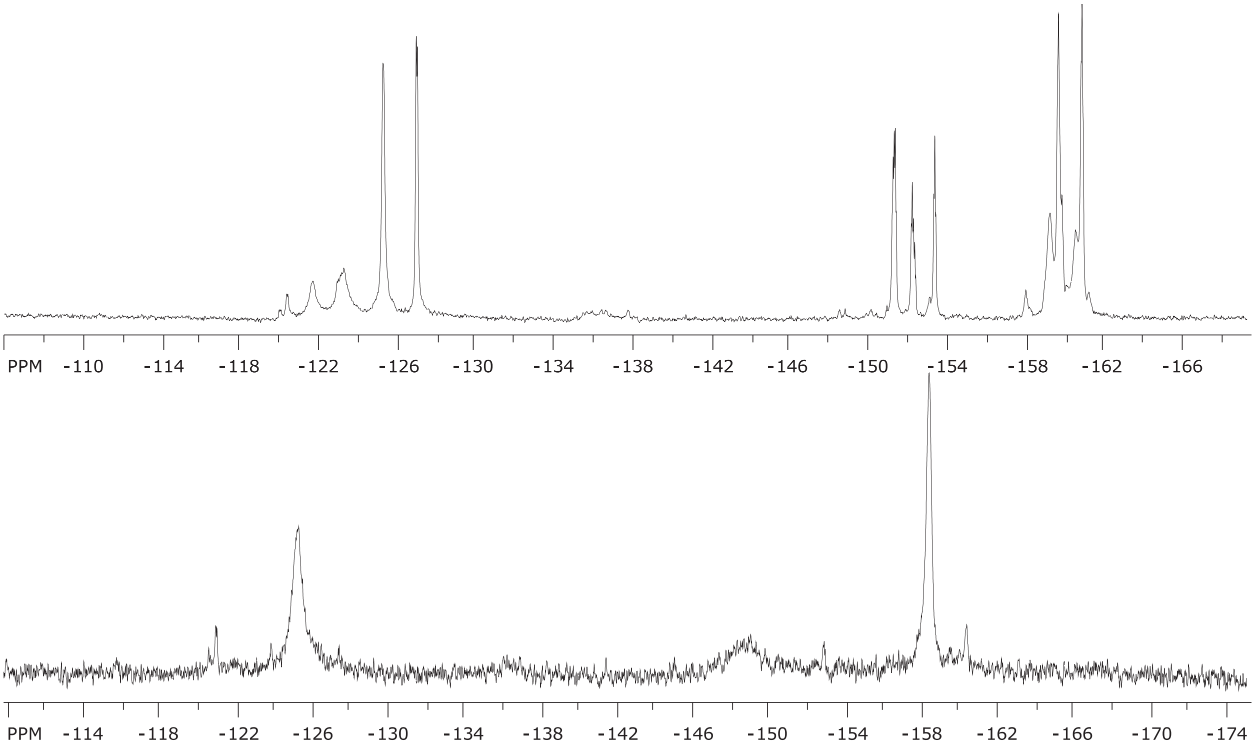

Figure S.32. ^{19}F NMR spectra in dichloromethane of biphasic test of 0.1% wt. aqueous formaldehyde (with Tritox-100 surfactant) after 90 min of sonication (top), compared with the same biphasic test containing only water (with surfactant) sonicated for the same amount of time (bottom) showing unreacted stiborane **7**.

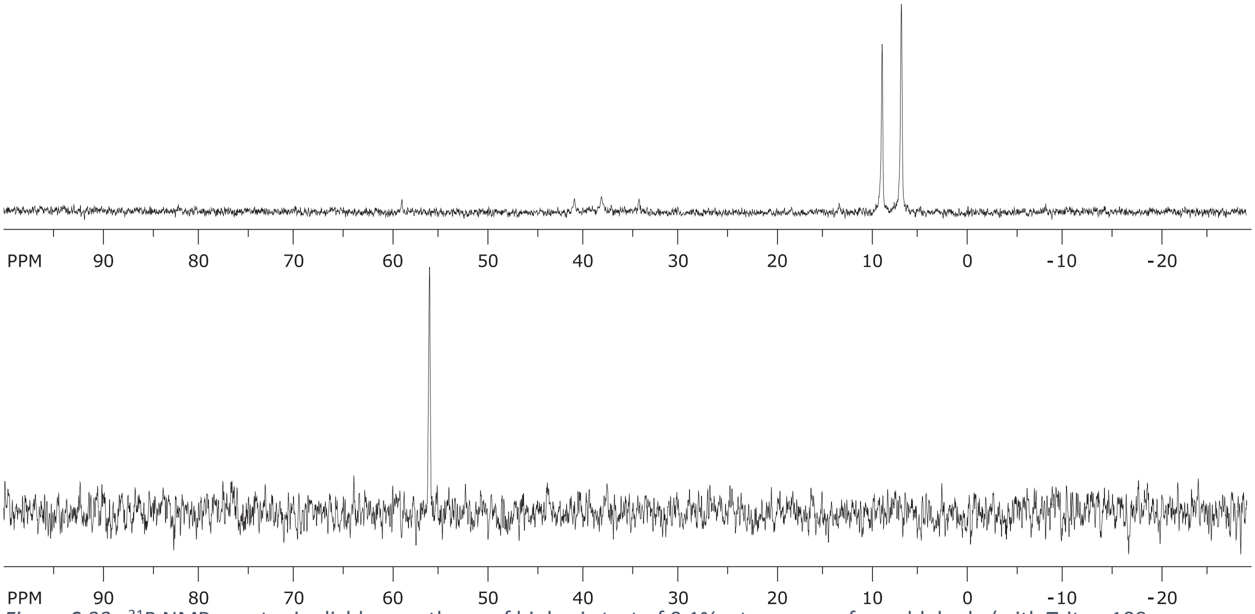

Figure S.33. ^{31}P NMR spectra in dichloromethane of biphasic test of 0.1% wt. aqueous formaldehyde (with Tritox-100 surfactant) after 90 min of sonication show the formation of two isomers 1:1 of **9** (top), compared to just unreacted stiborane **7** when the same test was performed with only water and surfactant (bottom).

S.11 Computational details

Table S.1. Summary of Sb–P distances (Å) in the computed in optimized geometries vs experimental solid-state structures. For the Et_3PO·**2** adduct, the Sb–$OPEt_3$ distance is given instead

Compound	Computed	Experimental	CSD No.
**7**	2.7691	2.8082(11)	1483469
**5**	2.96664	3.0268(12)	1483467
**6**	3.16919	3.2332(9)	1483468
**4**	3.31997	3.280(2)	—
Et_3PO·**2**	2.1014	2.107(2)	1483465

Table S.2. Optimized coordinates for stiborane **2**

Sb	10.538689	6.108551	-11.111218
O	12.276735	7.179756	-10.950466
O	10.848969	5.961243	-9.143444
C	12.706096	7.292025	-9.682946
C	11.943190	6.634398	-8.704401
C	12.309898	6.677439	-7.366693
C	13.457676	7.395139	-6.989965
C	14.221909	8.054439	-7.961920
C	13.844913	8.001584	-9.313832
Cl	11.332722	5.842827	-6.207033
Cl	13.916770	7.459657	-5.320190
Cl	15.638952	8.940912	-7.503151
Cl	14.757110	8.800080	-10.549273
C	11.440504	5.076499	-12.736083
F	9.444160	5.322569	-13.993309
C	10.716759	4.858963	-13.903227
F	10.535493	3.996658	-16.111075
C	11.255315	4.183726	-14.987353
C	12.554531	3.698459	-14.903532
F	13.091890	3.037737	-15.943047
F	14.545544	3.391476	-13.659419
C	13.293798	3.883489	-13.742724
F	13.468802	4.687425	-11.544010
C	12.732679	4.564547	-12.669892
C	8.999073	4.645979	-10.770747
F	7.188391	5.996342	-11.530535
C	7.645550	4.824446	-11.026733
F	5.408003	4.021478	-11.046791
C	6.716550	3.819392	-10.789636
F	6.250843	1.617697	-10.055805
C	7.142565	2.597307	-10.288177
F	8.907626	1.191793	-9.560019
C	8.491600	2.383499	-10.034350
F	10.714235	3.166117	-10.059021
C	9.396179	3.404357	-10.283551
C	9.533374	7.890232	-11.648141
F	8.321704	7.920660	-9.601404
C	8.636961	8.506315	-10.780180
F	7.180728	10.301236	-10.233844
C	8.048623	9.724653	-11.087766
F	7.801413	11.534100	-12.592595
C	8.365104	10.354258	-12.285004
F	9.577856	10.380867	-14.321268
C	9.266892	9.766755	-13.163288
F	10.732575	8.002821	-13.696183
C	9.843869	8.549407	-12.833000

Table S.3. Optimized coordinates for stiborane $SbPh_3(O_2C_6Cl_4)$

Sb	20.584290	16.032995	-11.153195	Cl	25.791407	18.738411	-7.437038
C	19.984854	18.599451	-12.717483	Cl	24.845621	18.784110	-10.466208
C	19.609216	17.848198	-11.595020	C	21.502690	15.052724	-12.790409
C	18.602423	18.310558	-10.735990	C	20.914496	13.878004	-13.283072
C	17.973786	19.529292	-11.003054	C	21.479511	13.226509	-14.382074
C	18.341850	20.276143	-12.124523	C	22.624096	13.744652	-14.990867
C	19.342768	19.810994	-12.981566	C	23.210699	14.913424	-14.499163
C	18.932321	14.731142	-10.769468	C	22.658225	15.569540	-13.397615
C	17.769643	14.890400	-11.540053	H	23.060390	13.237530	-15.843665
C	16.682896	14.031242	-11.355547	H	21.026279	12.316387	-14.757217
C	16.752567	13.009313	-10.406452	H	24.101663	15.314343	-14.968404
C	17.909649	12.844422	-9.641156	H	23.125233	16.464229	-13.008474
C	18.999307	13.700296	-9.819894	H	20.027094	13.465737	-12.817286
O	22.395018	17.125804	-10.924356	H	19.627019	20.390881	-13.851944
C	22.849392	17.159123	-9.678087	H	17.199435	19.891165	-10.336652
C	22.122687	16.411765	-8.725490	H	17.849694	21.219624	-12.330487
C	22.522990	16.380654	-7.396433	H	20.767349	18.249538	-13.381810
C	23.660971	17.100684	-6.989220	H	18.308991	17.732027	-9.866222
C	24.384431	17.844726	-7.927207	H	17.699731	15.680978	-12.280743
C	23.976748	17.872184	-9.272003	H	15.786419	14.162418	-11.950786
O	21.040257	15.754916	-9.180531	H	15.908292	12.344518	-10.263609
Cl	21.590587	15.444448	-6.270361	H	17.965217	12.051726	-8.903696
Cl	24.154664	17.062378	-5.324609	H	19.889487	13.569400	-9.217560

Table S.4. Optimized coordinates for the $Et_3PO\cdot$**2** adduct

Sb	-0.422275	-0.018207	-0.008007
P	-0.364698	2.682491	2.162434
O	-0.791359	1.311245	1.576989
C	0.065992	-1.532478	-1.539374
F	-0.392836	-0.116194	-3.406601
C	0.018989	-1.310025	-2.912764
F	0.303955	-1.995978	-5.173739
C	0.367484	-2.272628	-3.851340
F	1.122676	-4.470817	-4.324346
C	0.779312	-3.525673	-3.426921
F	1.207436	-5.022884	-1.641255
C	0.824557	-3.798249	-2.068988
F	0.511412	-3.177558	0.152184
C	0.469538	-2.813307	-1.155060
C	-1.691278	-1.412259	1.051750
F	-2.850851	-1.854532	-0.981159
C	-2.653388	-2.122713	0.340303
F	-4.382819	-3.750031	0.182486
C	-3.457815	-3.092913	0.916826
F	-4.081447	-4.317681	2.849697
C	-3.309114	-3.379723	2.266159
F	-2.219439	-2.961179	4.326448
C	-2.364051	-2.689796	3.009611
F	-0.677589	-1.093292	3.200476
C	-1.570476	-1.720655	2.403575
C	-1.873197	1.248491	-1.052407
F	-0.317854	2.287762	-2.572048
C	-1.569862	2.132109	-2.093613
F	-2.182483	3.773163	-3.698010
C	-2.532046	2.940565	-2.691838
F	-4.777922	3.690621	-2.829399
C	-3.845096	2.907440	-2.254543
F	-5.449327	2.040780	-0.739142
C	-4.179889	2.070489	-1.203013
F	-3.609278	0.523939	0.434541

C	-3.202049	1.274329	-0.622230
O	1.073507	1.224222	-0.634883
C	2.288535	0.803575	-0.242404
O	1.136400	-0.697912	1.134212
C	2.318574	-0.231616	0.717141
Cl	3.536084	-1.977506	2.367783
C	3.540498	-0.709907	1.185036
Cl	6.258435	-0.756224	1.296896
C	4.740466	-0.158054	0.704773
Cl	6.186781	1.554888	-0.844919
C	4.709274	0.867622	-0.246914
Cl	3.393693	2.615060	-1.902612
C	3.476263	1.346153	-0.723632
C	-0.377561	3.960398	0.883618
H	0.332808	3.646305	0.111279
H	-1.379387	3.925005	0.439095
C	-0.058273	5.376428	1.376070
H	-0.748896	5.705685	2.157685
H	-0.145386	6.077133	0.541169
H	0.961052	5.451493	1.763300
C	-1.589094	3.152991	3.413139
H	-1.112441	3.907691	4.050671
H	-2.396600	3.659747	2.869951
C	-2.158776	2.003676	4.249506
H	-2.669868	1.272446	3.619537
H	-2.887674	2.403029	4.960595
H	-1.385597	1.483756	4.818730
C	1.291613	2.639493	2.881553
H	1.979052	2.416074	2.056803
H	1.510097	3.666724	3.199599
C	1.493681	1.662575	4.042722
H	0.901798	1.948240	4.915975
H	2.546013	1.667403	4.340695
H	1.228686	0.641205	3.763059

Table S.5. Optimized coordinates for phosphino-stiborane **7**

Sb	-0.290096	0.437739	0.449897	F	-0.263539	3.166281	-1.427484
P	-1.195621	-2.167888	0.692063	C	-2.085891	0.969341	-0.670678
C	-1.180371	-0.072901	2.338693	C	-2.449989	0.416947	-1.895027
C	-1.550316	-1.428472	2.301917	C	-3.630413	0.767297	-2.540862
C	-2.121018	-2.033723	3.429463	C	-4.485894	1.693081	-1.96224
H	-2.391588	-3.083293	3.413053	C	-4.154643	2.263187	-0.741526
C	-2.335934	-1.264665	4.573209	C	-2.970404	1.891637	-0.122214
H	-2.773556	-1.721366	5.452822	F	-1.670347	-0.502141	-2.510866
C	-1.994693	0.092619	4.592443	F	-3.955004	0.207939	-3.728161
H	-2.173918	0.679102	5.485979	F	-5.634576	2.034146	-2.58049
C	-1.414616	0.700953	3.474907	F	-4.98181	3.168201	-0.171055
H	-1.149388	1.749908	3.506381	F	-2.676897	2.483362	1.071006
O	0.661564	0.250904	-1.348914	C	-0.233112	-3.683337	0.957353
O	1.542298	-0.330463	1.062625	C	1.01409	-3.564113	1.596862
C	1.932532	-0.166311	-1.244086	H	1.380762	-2.587211	1.890604
C	2.408817	-0.467777	0.051483	C	1.775608	-4.701542	1.85733
C	3.72544	-0.889574	0.222591	H	2.732828	-4.6029	2.35573
C	4.573649	-1.028557	-0.889348	C	1.309673	-5.962391	1.474388
C	4.096925	-0.738765	-2.172164	H	1.906418	-6.844806	1.673614
C	2.771989	-0.302671	-2.345713	C	0.073611	-6.084245	0.837678
Cl	4.282703	-1.220815	1.832018	H	-0.293975	-7.060395	0.543859
Cl	6.211373	-1.55871	-0.661594	C	-0.700451	-4.951651	0.579715
Cl	5.133323	-0.912294	-3.55409	H	-1.66283	-5.062933	0.09675
Cl	2.142063	0.079706	-3.916014	C	-2.755612	-2.627677	-0.105244
C	0.488875	2.446386	0.723722	C	-2.718956	-3.195364	-1.392376
C	1.191316	2.802016	1.871893	H	-1.768909	-3.393129	-1.876422
C	1.763078	4.056658	2.040153	C	-3.906707	-3.496895	-2.056907
C	1.642329	5.003278	1.033803	H	-3.870119	-3.936527	-3.046503
C	0.95379	4.684422	-0.127314	C	-5.138145	-3.225521	-1.453659
C	0.394163	3.421023	-0.266463	H	-6.059355	-3.456765	-1.975498
F	1.344174	1.917255	2.890152	C	-5.179897	-2.65074	-0.182145
F	2.435693	4.360999	3.172889	H	-6.132268	-2.436117	0.288377
F	2.191533	6.225194	1.182672	C	-3.995415	-2.351239	0.493415
F	0.836788	5.602458	-1.112813	H	-4.041254	-1.905081	1.479457

Table S.6. Optimized coordinates for phosphino-stiborane **5**

Sb	0.896432	-1.190466	-0.250896	H	2.445743	-3.833047	0.444102
P	0.95937	1.775054	-0.302937	C	2.4653	-1.227464	1.203128
O	-0.437586	-1.160244	1.332809	C	3.762261	-0.877634	0.79968
O	-0.921609	-0.742706	-1.210292	H	3.954827	-0.550573	-0.21688
C	1.861856	-0.19479	-1.888612	C	4.822065	-0.939974	1.709324
C	1.820648	1.209225	-1.804208	H	5.820998	-0.66343	1.391395
C	2.391187	1.978266	-2.828581	C	4.591824	-1.352126	3.023358
H	2.341503	3.060333	-2.797845	H	5.413121	-1.397643	3.729636
C	3.012423	1.342951	-3.905695	C	3.301569	-1.704729	3.428213
H	3.452697	1.937685	-4.697622	H	3.120988	-2.026101	4.44799
C	3.064773	-0.05256	-3.972835	C	2.238028	-1.643531	2.524447
H	3.546465	-0.535358	-4.815372	H	1.240843	-1.914333	2.847045
C	2.486999	-0.83037	-2.964423	C	-0.251089	3.026956	-0.840581
H	2.51845	-1.912647	-3.027708	C	-0.699941	3.995877	0.074019
Cl	-2.340392	-0.967384	3.608665	H	-0.268939	4.046865	1.067845
Cl	-5.279258	-0.194073	2.694887	C	-1.69884	4.898704	-0.290006
Cl	-5.875608	0.273534	-0.367333	H	-2.035511	5.641837	0.423768
Cl	-3.536215	-0.057527	-2.480172	C	-2.262909	4.846749	-1.56723
C	-1.688349	-0.831302	0.99996	H	-3.038822	5.549222	-1.8486
C	-1.951271	-0.614661	-0.374775	C	-1.823105	3.885771	-2.480425
C	-3.241345	-0.281424	-0.783528	H	-2.255649	3.840272	-3.473455
C	-4.274432	-0.147981	0.160644	C	-0.82716	2.976122	-2.120813
C	-4.010585	-0.353647	1.518096	H	-0.501558	2.230206	-2.836083
C	-2.711813	-0.695698	1.933606	C	2.128837	2.70242	0.746681
C	0.791868	-3.235059	-0.81949	C	2.151835	2.380864	2.113594
C	-0.162024	-3.672241	-1.752225	H	1.503683	1.598662	2.493428
H	-0.867023	-2.96863	-2.178645	C	3.006374	3.060384	2.984605
C	-0.203864	-5.016162	-2.130711	H	3.015041	2.804802	4.037768
H	-0.943864	-5.348909	-2.849833	C	3.849554	4.059473	2.496696
C	0.702103	-5.929059	-1.584933	H	4.517092	4.583682	3.171043
H	0.665064	-6.971754	-1.879337	C	3.832949	4.385422	1.136903
C	1.654755	-5.498091	-0.65962	H	4.485581	5.163113	0.756872
H	2.359211	-6.203838	-0.234055	C	2.974771	3.71575	0.265355
C	1.702344	-4.154981	-0.277076	H	2.964497	3.985127	-0.783921

Table S.7. Optimized coordinates for phosphino-stibine **6**

Sb	0.517212	-0.550390	-0.621600
P	-2.612981	-0.283376	-0.204056
C	-1.801099	-0.811547	1.349174
C	-0.391535	-0.907572	1.305196
C	0.309530	-1.313076	2.445994
H	1.388876	-1.407256	2.427956
C	-0.376299	-1.581423	3.635476
H	0.177841	-1.877268	4.519013
C	-1.764451	-1.453631	3.689052
H	-2.294038	-1.650593	4.614095
C	-2.476181	-1.074241	2.548252
H	-3.555300	-0.977125	2.593286
C	-3.127600	-1.846431	-1.002789
C	-3.112896	-3.098245	-0.368756
H	-2.825309	-3.176332	0.673327
C	-3.462997	-4.251273	-1.076570
H	-3.444350	-5.213448	-0.577224
C	-3.835354	-4.166645	-2.419652
H	-4.103807	-5.063084	-2.966832
C	-3.852129	-2.923985	-3.059993
H	-4.133473	-2.853516	-4.104552
C	-3.492302	-1.772985	-2.359782
H	-3.487210	-0.813057	-2.867315
C	-4.178952	0.496420	0.327946
C	-4.141671	1.861481	0.663844
H	-3.204867	2.405993	0.605031
C	-5.301333	2.521806	1.068524
H	-5.259447	3.573448	1.328359

C	-6.515502	1.832319	1.126760
H	-7.418549	2.348392	1.432224
C	-6.564138	0.479499	0.784002
H	-7.504722	-0.058197	0.825433
C	-5.402922	-0.188354	0.388768
H	-5.449766	-1.238959	0.125817
C	0.770906	1.615971	-0.592935
F	1.874156	1.353290	-2.685895
C	1.395285	2.172454	-1.704420
F	2.191819	4.032254	-2.967617
C	1.572665	3.538715	-1.870698
F	1.261292	5.736267	-1.031693
C	1.103061	4.403348	-0.892546
F	0.017638	4.731349	1.188270
C	0.472327	3.888863	0.231322
F	-0.297625	2.079727	1.496067
C	0.315255	2.514521	0.365676
C	2.573486	-0.960658	0.042993
F	2.451952	-2.960693	-1.246537
C	3.168685	-2.119023	-0.446756
F	5.013905	-3.617347	-0.654649
C	4.477328	-2.479526	-0.156965
F	6.515523	-1.983774	0.951244
C	5.240875	-1.653956	0.656393
F	5.426788	0.310731	1.969114
C	4.685310	-0.490016	1.168621
F	2.876536	0.975371	1.414463
C	3.369838	-0.164980	0.860355

Table S.8. Optimized coordinates for phosphino-stibine **4**

Sb	0.517212	-0.55039	-0.6216
P	-2.612981	-0.283376	-0.204056
C	-1.801099	-0.811547	1.349174
C	-0.391535	-0.907572	1.305196
C	0.30953	-1.313076	2.445994
H	1.388876	-1.407256	2.427956
C	-0.376299	-1.581423	3.635476
H	0.177841	-1.877268	4.519013
C	-1.764451	-1.453631	3.689052
H	-2.294038	-1.650593	4.614095
C	-2.476181	-1.074241	2.548252
H	-3.5553	-0.977125	2.593286
C	-3.1276	-1.846431	-1.002789
C	-3.112896	-3.098245	-0.368756
H	-2.825309	-3.176332	0.673327
C	-3.462997	-4.251273	-1.07657
H	-3.44435	-5.213448	-0.577224
C	-3.835354	-4.166645	-2.419652
H	-4.103807	-5.063084	-2.966832
C	-3.852129	-2.923985	-3.059993
H	-4.133473	-2.853516	-4.104552
C	-3.492302	-1.772985	-2.359782
H	-3.48721	-0.813057	-2.867315
C	-4.178952	0.49642	0.327946
C	-4.141671	1.861481	0.663844
H	-3.204867	2.405993	0.605031
C	-5.301333	2.521806	1.068524
H	-5.259447	3.573448	1.328359

C	-6.515502	1.832319	1.12676
H	-7.418549	2.348392	1.432224
C	-6.564138	0.479499	0.784002
H	-7.504722	-0.058197	0.825433
C	-5.402922	-0.188354	0.388768
H	-5.449766	-1.238959	0.125817
C	0.770906	1.615971	-0.592935
F	1.874156	1.35329	-2.685895
C	1.395285	2.172454	-1.70442
F	2.191819	4.032254	-2.967617
C	1.572665	3.538715	-1.870698
F	1.261292	5.736267	-1.031693
C	1.103061	4.403348	-0.892546
F	0.017638	4.731349	1.18827
C	0.472327	3.888863	0.231322
F	-0.297625	2.079727	1.496067
C	0.315255	2.514521	0.365676
C	2.573486	-0.960658	0.042993
F	2.451952	-2.960693	-1.246537
C	3.168685	-2.119023	-0.446756
F	5.013905	-3.617347	-0.654649
C	4.477328	-2.479526	-0.156965
F	6.515523	-1.983774	0.951244
C	5.240875	-1.653956	0.656393
F	5.426788	0.310731	1.969114
C	4.68531	-0.490016	1.168621
F	2.876536	0.975371	1.414463
C	3.369838	-0.16498	0.860355
